# miR168 targets Argonaute1A mediated miRNAs regulation pathways in response to potassium deficiency stress in tomato

**DOI:** 10.1186/s12870-020-02660-5

**Published:** 2020-10-19

**Authors:** Xin Liu, Chunchang Tan, Xin Cheng, Xiaoming Zhao, Tianlai Li, Jing Jiang

**Affiliations:** 1 Horticulture Department, College of Horticulture, Shenyang Agricultural University, No. 120 Dongling Road, Shenhe District, Shenyang, 110866 P.R. China; 2 Key Laboratory of Protected Horticulture of Ministry of Education, No. 120 Dongling Road, Shenhe District, Shenyang, 110866 P.R. China; 3 Key Laboratory of Protected Horticulture of Liaoning Province, No. 120 Dongling Road, Shenhe District, Shenyang, 110866 P.R. China

**Keywords:** Argonaute1, *miR168*, microRNA, Potassium deficiency stress, Root, Target genes

## Abstract

**Background:**

Potassium (K^+^) is an essential ion for most plants, as it is involved in the regulation of growth and development. K^+^ homeostasis in plant cells has evolved to facilitate plant adaptation to K^+^-deficiency stress. Argonaute1 (AGO1) is regulated by *miR168* to modulate the small RNA regulatory pathway by RNA silencing complex (RISC) in tomatoes. However, the role of *miR168*-mediated regulation of AGO1 in the context of K^+^ deficiency stress in tomatoes has not been elucidated yet.

**Results:**

*SlmiR168* and its target gene *SlAGO1A* were differentially expressed among low-K^+^-tolerant JZ34 and low-K^+^-sensitive JZ18 tomato plants. Transgenic tomato plants constitutively expressing *pri-SlmiR168a* showed stronger root system growth, better leaves development, and higher K^+^ contents in roots under K^+^-deficiency stress than those of the transgenic tomato lines expressing *rSlAGO1A* (*SlmiR168*-resistant) and the wild type (WT). Deep sequencing analysis showed that 62 known microRNAs (miRNAs) were up-regulated in *35S:rSlAGO1* compared with WT tomatoes. The same miRNAs were down-regulated in *35S:SlmiR168a* compared with WT plants. The integrated analysis found 12 miRNA/mRNA pairs from the 62 miRNAs, including the root growth and cytokinin (CTK)/abscisic acid (ABA) pathways.

**Conclusions:**

The regulation mediated by *SlmiR168* of *SlAGO1A* contributes to the plant development under low-K^+^ stress. Moreover, this regulation mechanism may influence downstream miRNA pathways in response to low-K^+^ stress through the CTK/ABA and root growth modulation pathways.

## Background

Macronutrients and micronutrients are the elements required necessarily for growth and development of plants [1]. N (Nitrogen), P (Phosphorus) and K (Potassium) were needed at relatively large amounts for plants. Phosphorus deficiency could reduces the plant growth and biomass production [2, 3]. It is more important especially in tomato production systems, which require substantial inputs of nitrogen fertilizer [4]. K^+^ is also a kind of the essential macronutrients that is involved in many physiological processes in plant cells, such as osmoregulation, ion homeostasis, photosynthesis, membrane potential maintenance, cell turgor, and others [5]. These functions rely on a high and relatively stable concentration of K^+^ in cellular compartments and K^+^ movement between different cellular compartments, cells, and tissues. Accordingly, K^+^ must be readily transported and K^+^ flow must be tightly regulated. In the soil, K^+^ is taken up by plants through root absorption. The K^+^ concentration in the cytoplasm is generally maintained at approximately 100 mM [6]. Compared with the high K^+^ concentration in cells, the concentration of K^+^ in the soil is very low. Moreover, because of the direct contact with the soil by roots of the plant, K^+^ deficiency signal is first perceived by root cells, particularly root epidermal cells and root hair cells [7]. Plants respond to K^+^ deficiency by altering root growth and root configuration, such as inhibiting primary roots and stimulating root hair elongation [8]. We described previously that the low-K^+^ tolerant JZ34 and low-K^+^ sensitive JZ18 tomato genotypes display different root configurations under K^+^-deficiency stress [9].

Low K^+^ stress can excite the activity of many signaling molecules, including reactive oxygen species (ROS), Ca^2+^, plant hormones, and microRNAs (miRNAs) in plant cells [10]. In *Arabidopsis*, K^+^ deficiency induces ROS production and the expression of the NADPH oxidase gene *RHD2* and the peroxidation enzyme gene *RCI3* [11]. In addition to ROS, Ca^2+^ also acts as a low K^+^ response signal. Ca^2+^ sensors (CBL1 and CBL9) participate in the low-K^+^ response and their target protein kinase CIPK23 also interact with AKT1 for K^+^ absolution [12]. Moreover, the Ca^2+^ reporter YC3.6 can also be induced by low K^+^ stress [9]. Many phytohormones signal transduction pathways are involved in response to K^+^-deficiency stress, such as ethylene, auxin, cytokinin (CTK), and abscisic acid (ABA). It was reported that ethylene production under K^+^-deficiency stress is upregulated [11, 13]. Moreover, ethylene signaling can also regulate *AtHAK5* transcription and root growth in *Arabidopsis thaliana* [14]. In addition, low-K^+^ treatment reduced the auxin accumulation by decreasing the auxin transporter AtPIN1 protein [15]. The K^+^ transporter AtTRH1/AtKUP is also regulated by the localization of AtPIN1 and influences K^+^-dependent root architecture in *A. thaliana* [16, 17]. Low-K^+^ stress also induces OsHAK16p:WOX11, an integrator of auxin and cytokinin signaling, resulting in enhanced root growth and development [18]. CTK accumulation decreases under low-K^+^ stress, with a concomitant increased ROS production [19]. A previous study suggested that the inhibitory effect of ABA on K^+^ uptake might be related to K^+^-ATPase [20]. The addition of 5 μM ABA inhibited the transport of K^+^ under low-K^+^ stress [21]. Collectively, these data suggest that phytohormone signaling pathways could synergistically regulate root morphology and K^+^ transport or accumulation under low-K^+^ stress conditions.

Transcriptional regulation that act as key roles, eventually mediating downstream plant responses, particularly under stress conditions [22]. miRNAs as the post-transcriptional factors, were few investigated in the role response to the K^+^ deficiency, compared to other nutrient elements [23, 24]. It has been shown that the miR444/MADS-box model, as well as pathways mediated by miR319/TCP4 and miR396/GRF, may contribute to low-K^+^ tolerance in barley plants [25]. In *O. sativa*, *miR399* is induced by low-K^+^ stress [23]. We previously found that the JZ18 and JZ34 tomato genotypes have a different miRNAs expression pattern under K^+^ deficiency stress, as determined by miRNA-seq. In particular, we found that miRNA168 was significantly differentially expressed in both JZ18 and JZ34 tomato genotypes under K^+^ deficiency stress, and validated that, in tomato; Actually, miRNA168 was confirmed to target the *Argonaute1* (*AGO1*) in tomato [26]. In plants, after RNase III Dicer-like 1 cutting, the miRNA strand of the miRNA: miRNA* duplex is loaded into an AGO protein, which has a single-stranded RNA-binding PAZ domain and an RNaseH-like PIWI domain to catalyze mRNA cleavage or translational repression [27, 28]. The AGO protein is a core element of the RNA induced silencing complex (RISC), a transcriptional and post-transcriptional regulator that is guided by small RNAs to repress target genes expression. MiRNAs are loaded into AGO1, which acts as an RNA slicer [29]. Fifteen *SlAGO* genes were detected in tomato [30]. *SlAGO1A*, *SlAGO1B*, and *SlAGO2A* are targeted by conserved miRNAs [30]. In *Arabidopsis*, the fine-tuned post-transcriptional regulation of *miR168* and *AGO1* levels maintains the homeostasis of other miRNAs combined with AGO1, control the target genes expression levels of miRNA [31]. Kidner and Martienssen reported that *ago1* showed the leaf polarity defect which was caused by an abnormal distribution of miRNAs and their targets are known to control leaf polarity [32]. Moreover, it has been shown that the steady-state levels of several transcription factors targeted by miRNAs are increased in leaves of *ago1* plants [33]. AGO proteins levels are also crucial in virus defense: overexpression of AGO proteins induces plant development disorder during virus infection [34]. The interaction of AC2 with AGO1 was confirmed after Tomato leaf curl New Delhi virus [35]. In addition, miR168a regulating SlAGO1s affected the pathogenesis-related genes in tomato plants to change the resistance to disease [36].

In this study, we aimed to evaluate the balance between *SlmiR168* and *SlAGO1 (SlAGO1A*) expression in response to K^+^ deficiency stress in *Solanum lycopersicum*. We characterized the plant phenotype in response to low-K^+^ stress conferred by *35S:SlmiR168a* and *35S:rSlAGO1*. We discovered that *35S:SlmiR168a* plants had a stronger root system and better leaf development than those of *35S:rSlAGO1* plants under low-K^+^ stress. This prompted us to use miRNA-Seq and mRNA-Seq to assess the miRNAs potential regulatory mechanism of *SlmiR168*-mediated regulation of *SlAGO1A* in response to K^+^ deficiency.

## Results

### Differential expression of *SlmiR168* and *SlAGO1A* in JZ18 and JZ34

**Fig. 1:**
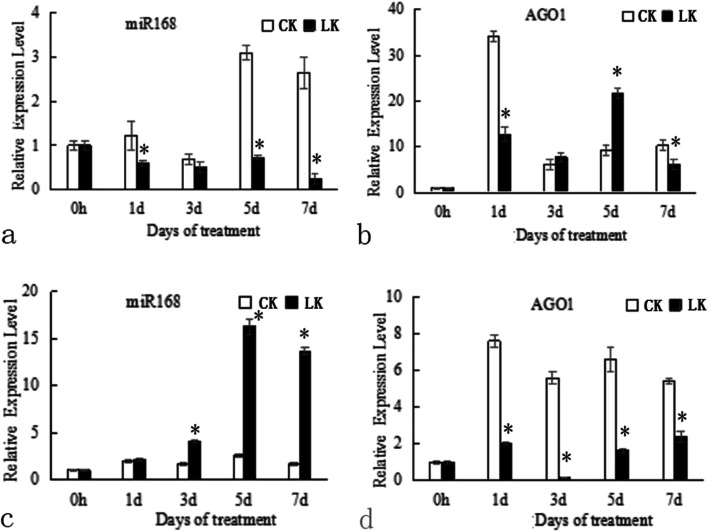
*SlmiR168* and *SlAGO1A* expression profiles in JZ18 and JZ34 plants under normal K^+^ conditions and K^+^ deficiency conditions. Samples of leaves were collected at 0, 1, 3, 5, and 7 days after treatment. **a**. the miR168 expression levels in JZ18 seedlings under normal K^+^ and K^+^ deficiency conditions; **b**. the *SlAGO1A* expression levels in JZ18 seedlings under normal K^+^ and K^+^ deficiency conditions; **c**. the miR168 expression levels in JZ34 seedlings under normal K^+^ and K^+^ deficiency conditions; **d**. the *SlAGO1A* expression levels in JZ34 seedlings under normal K^+^ and K^+^ deficiency conditions. CK: normal K^+^ (4 mM); LK: K^+^ deficiency (0.5 mM). Error bars indicate the means ± SE of three independent replicates. * Significant differences with *P* < 0.05 determined using a Duncan’s test compared with the CK

JZ34 tomatoes (low-K^+^-tolerant), compared to JZ18 tomatoes (low-K^+^-sensitive), show better root development and K^+^ absorption under K^+^ deficiency conditions [9]. According to our previous study, *SlmiR168* is differentially expressed between JZ18 and JZ34 tomatoes under K^+^ deficiency stress. In this study, we first performed quantitative reverse transcription PCR (RT-PCR) and observed that the expression levels of *SlmiR168* increased with time in JZ18 tomatoes under normal conditions. However, under K^+^ deficiency stress, the *SlmiR168* levels decreased in a time-dependent manner (Fig. 1a). In contrast, in JZ34 tomatoes, the expression levels of *SlmiR168* significantly increased under K^+^ deficiency stress, particularly after 3, 5, and 7 days of treatment (Fig. 1c). This expression pattern suggested that *SlmiR168* expression might be involved in the regulation of tomatoes tolerance to K^+^ deficiency. The expression levels of the target gene, *SlAGO1A,* showed a complementary pattern. *SlAGO1A* expression levels were up-regulated after K^+^ deficiency stress treatment for 3 and 5 days in JZ18 (Fig. 1b). The target *AGO1A* expression levels were obviously decreased under K^+^ deficiency compared with that under normal conditions in JZ34 tomatoes (Fig. 1d). As a target of *SlmiR168*, *SlAGO1A* showed a complementary expression pattern, and the expression of *SlmiR168* actually responded to the low K^+^ stress. The differential expression patterns of *SlmiR168* and *SlAGO1A* between JZ18 and JZ34 tomatoes may be a cause of the variations in tolerance of the two tomato genotypes under low K^+^ stress.

### Analysis of *SlmiR168* and *SlAGO1A* expression in different tissues

**Fig. 2:**
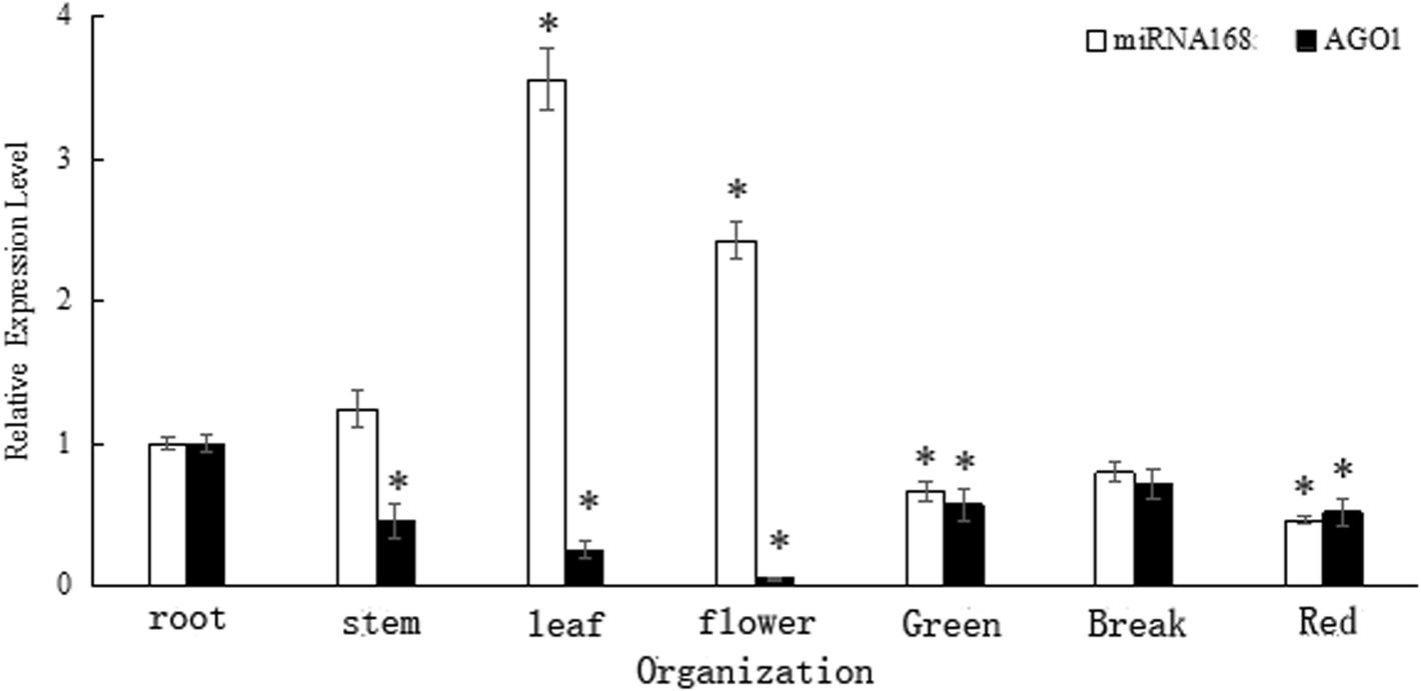
*SlmiR168* and *SlAGO1A* expression profiles in different ‘JZ18’ tomato tissues (roots, stems, leaves, flowers, green fruits, break fruits and red fruits). The tomatoes were grown in the normal conditions. Error bars indicate the means ± SE of three independent replicates. * Significant differences with *P* < 0.05 determined using a Duncan’s test compared with the control

The expression of *SlmiR168* and *SlAGO1A* in different tissues of tomato plants was evaluated by RT-PCR (Fig. 2). *SlmiR168* and *SlAGO1A* were detected in all tissues. The expression levels of *SlmiR168* were highest in the leaves and flowers, followed by the roots and stems. Conversely, in the stems, leaves, and flowers, *SlAGO1A* showed the opposite expression pattern.

### Regulation of *SlAGO1A* by *SlmiR168* increases plant tolerance to K^+^ deficiency stress

**Fig. 3:**
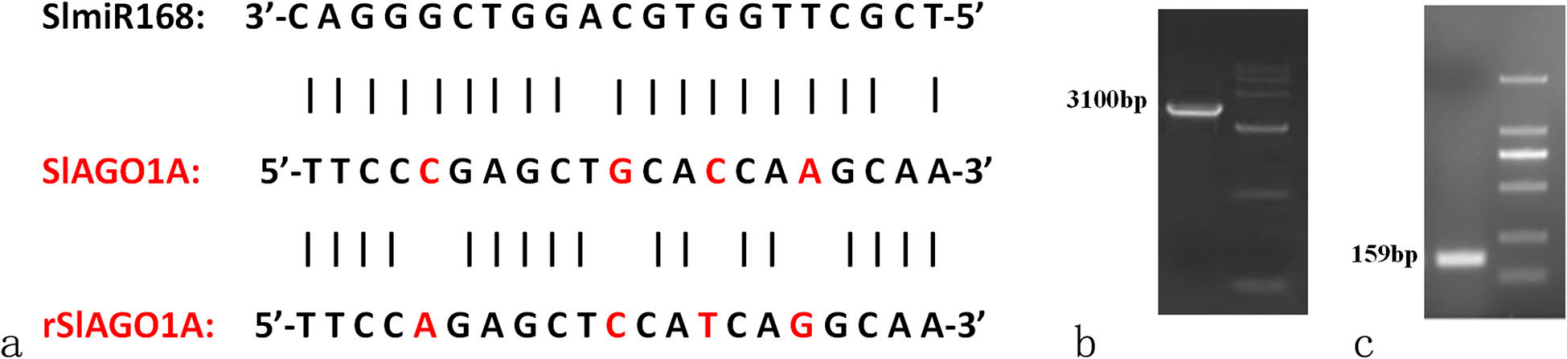
Mutant *SlAGO1A* transgenic transcripts were resistant to *SlmiR168*-mediated cleavage in tomatoes. **a**. Representation of the constructs used for transgenic expression of the *SlmiR168*-resistant mutant (*rSlAGO1A*) in tomatoes. Mutations were introduced at four locations (in red), and these base changes did not affect the native protein sequence. **b**. Amplification of the *rSlAGO1* cDNA band located at 3100 bp. **c**. Amplification of the *pri-SlmiR168a* band at 159 bp

**Fig. 4:**
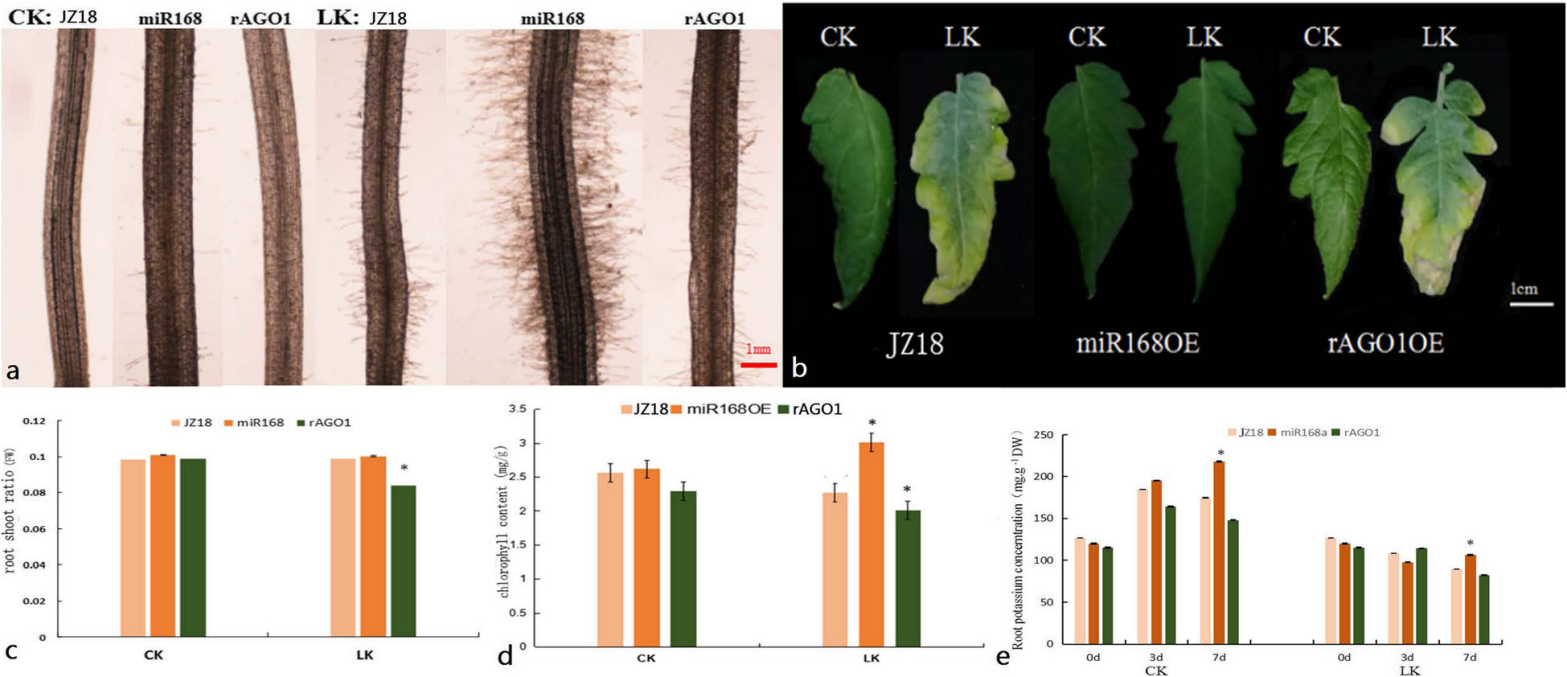
Comparison of morphological changes in WT, *35S:SlmiR168a* ‘Line 4’, and *35S:rSlAGO1* ‘Line 2’ plants under normal K^+^ conditions and K^+^ deficiency stress after 7 days of treatment. **a**. Changes in the root hair region in WT, *35S:SlmiR168a*, and *35S:rSlAGO1* plants under CK and LK conditions after 7 days (100 × magnification) with three biological replicates. **b**. Differences in the fifth leaf from the bottom up in WT, *35S:SlmiR168a*, and *35S:rSlAGO1* plants under CK and LK conditions after 15 days. **c**. Root-shoot ratios in WT, *35S:SlmiR168a*, and *35S:rSlAGO1* plants under CK and LK conditions after 7 days with three biological replicates. * Significant differences with P < 0.05 determined using a Duncan’s test compared with the CK. **d**. Chlorophyll contents of the leaves in WT, *35S:SlmiR168a*, and *35S:rSlAGO1* plants under CK and LK conditions after 7 days with three biological replicates.* Significant differences with P < 0.05 determined using a Duncan’s test compared with the CK. **e**. K^+^ contents of the roots in WT, *35S:SlmiR168a*, and *35S:rSlAGO1* plants under CK and LK conditions after 3 and 7 days with three biological replicates. * Significant differences with P < 0.05 determined using a Duncan’s test compared with the CK. CK: normal K^+^ (4 mM); LK: K^+^ deficiency (0.5 mM)

To elucidate whether the regulation of *SlAGO1* by *SlmiR168a* was responsible for differences in low K^+^ tolerance, 37 transformants of *35S:SlmiR168a* and 6 transformants of *35S:rSlAGO1* were obtained. The expression levels of *SlmiR168* and the target gene *SlAGO1A* of transformants were list in supporting files (Fig. S8 a and b). *SlmiR168*-resistant constructs (*rSlAGO1A*) were generated using the point mutation (Fig. 3a), which did not change the native protein sequence of *SlAGO1* by mutation of four bases. Furthermore, *rSlAGO1A* can’t be regulated by *SlmiR168*, so *35S:rSlAGO1* transformants could illustrate the role of the regulation of *SlmiR168*. The *rSlAGO1* and *pri-SlmiR168a* fragments were amplified by PCR for overexpression vector construction (Fig. 3b and c; Referring to the original figures: Figs. S6 and S7); *35S:SlmiR168a* ‘Line 4’and *35S:rSlAGO1* ‘Line 2′ transformants were selected for experiments. The root morphology of WT, *35S:SlmiR168a*, and *35S:rSlAGO1* all showed a larger root system after 7 days of development at the normal K^+^ concentration (4 mM) (Fig. S5). However, in the presence of low K^+^ concentration (0.5 mM), the roots of *35S:SlmiR168a* plants appeared stronger than WT and *35S:rSlAGO1* plants after 7 days of treatment. Microexamination revealed that the number of root hairs was obviously increased in *35S:SlmiR168a* plants following low K^+^ treatment at 7 days compared with that in WT and *35S:rSlAGO1* plants (Fig. 4a). Leaf development was also observed under K^+^ deficiency stress (Fig. 4b). The leaf margins of WT plants turned yellow under low K^+^ conditions, and those of *35S:rSlAGO1* plants showed increased yellowing; in contrast, *35S:SlmiR168a* plants did not exhibit yellowing of the leaves. Analysis of the root-shoot ratio (Fig. 4c) showed that under low K^+^ conditions, root-shoot ratios of *35S:SlmiR168a* plants did not differ significantly compared with that in WT plants. However, *35S:rSlAGO1* plants exhibited a decreased root-shoot ratio compared with WT and *35S:SlmiR168a* plants. Additionally, the chlorophyll content was highly increased in *35S:SlmiR168a* plants but decreased in WT and *35S:rSlAGO1* plants under K^+^ deficiency stress compared with the normal K^+^ condition (Fig. 4d). Chlorophyll contents in *35S:rSlAGO1* plants were significantly lower than WT plants under low K^+^ conditions (Fig. 4d). Chlorophyll contents in *35S:SlmiR168a* plants were significantly higher than WT plants under low K^+^ conditions (Fig. 4d). Analysis of K^+^ contents in roots (Fig. 4e) showed that under normal K^+^ concentration conditions, *35S:SlmiR168a* plants showed increased K^+^ contents with development time, particularly reached the peak at 7 days of treatment. Under K^+^ deficiency stress, *35S:SlmiR168a* plants also showed significantly higher K^+^ contents than WT plants at 7 days treatment, whereas *35S:rSlAGO1* plants exhibited a little lower K^+^ contents than WT plants at 7 days treatment. Under low K^+^ concentration conditions, *35S:SlmiR168a* plants exhibited improved root and leaf growth under K^+^ deficiency stress. Moreover, *35S:SlmiR168a* plants exhibited higher K^+^ contents in roots under K^+^ deficiency stress. So *35S:SlmiR168a* plants demonstrated the better tolerance to K^+^ deficiency stress than *35S:rSlAGO1* and WT plants .

### Analysis of miRNA sequencing data in *35S:SlmiR168a* and *35S:rSlAGO1* plants

**Fig. 5:**
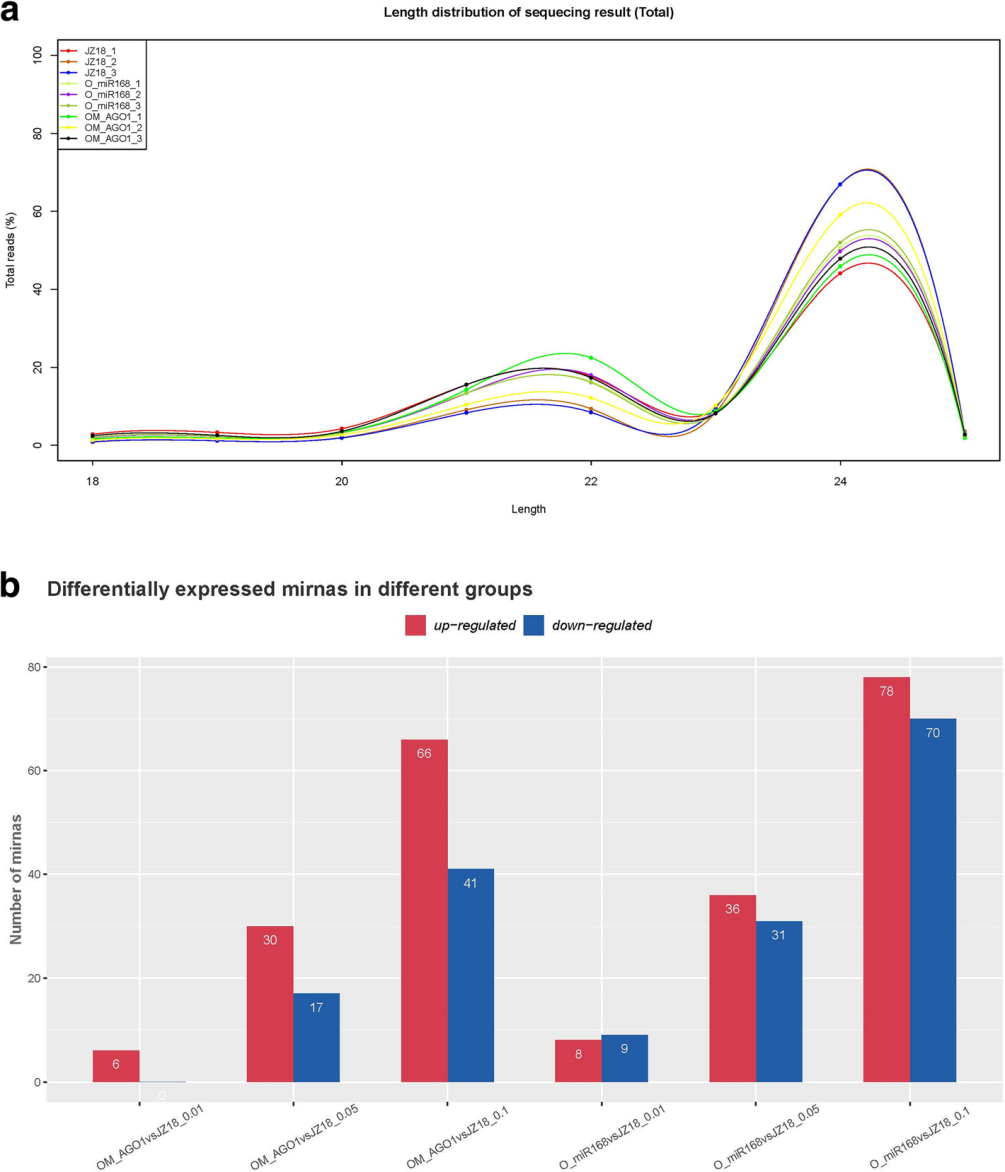
Differentially expressed miRNAs in WT, *35S:SlmiR168a* ‘Line 4’, and *35S:rSlAGO1* ‘Line 2’ plants. **a**. Length distribution of total identified miRNAs. **b**. Numbers of differetially expressed miRNAs in *35S:rSlAGO1* plants compared with WT and *35S:SlmiR168a* plants compared with WT. P < 0.05, 0.01, or 0.001

To identify miRNAs regulated by *SlmiR168*-mediated *SlAGO1A* in response to K^+^ deficiency stress, nine small RNA libraries were constructed from WT, *35S:SlmiR168a*, and *35S:rSlAGO1* samples. In total, 12,836,013, 14,373,027, 13,912,496, 14,850,199, 17,821,390, 12,006,556, 17,470,288, 12,383,616, and 25,030,158 raw reads were generated by high-throughput sequencing for the three kinds of samples and three replicates (Table S2). After data processing, including filtration of small RNAs except miRNAs, 7,163,035, 11,223,930, 9,849,836, 8,542,869, 10,694,993, 7,571,073, 10,723,320, 9,305,655, and 16,653,370 total valid reads, corresponding to 2,575,545, 4,694,297, 4,410,072, 3,159,839, 3,691,188, 2,895,817, 3,862,724, 4,199,533, and 53,931,63 unique reads were acquired in the libraries of WT, *35S:SlmiR168a*, and *35S:rSlAGO1* plants (with three replicates each), respectively. The most valid reads were 20–24 nt in length, with 24-nt reads being the most common among all three genotypes (Fig. 5a). Totally, 1168 conserved miRNAs and 1060 predicted novel miRNAs were identified in the nine small RNA libraries (Table S3). Details about family members of conserved miRNA are list in Table S4. Overall, 68 conserved miRNA families were contained in all the differentially expressed miRNAs.

### Analysis of miRNAs differential expressed in the two transgenic tomato plants

**Fig. 6:**
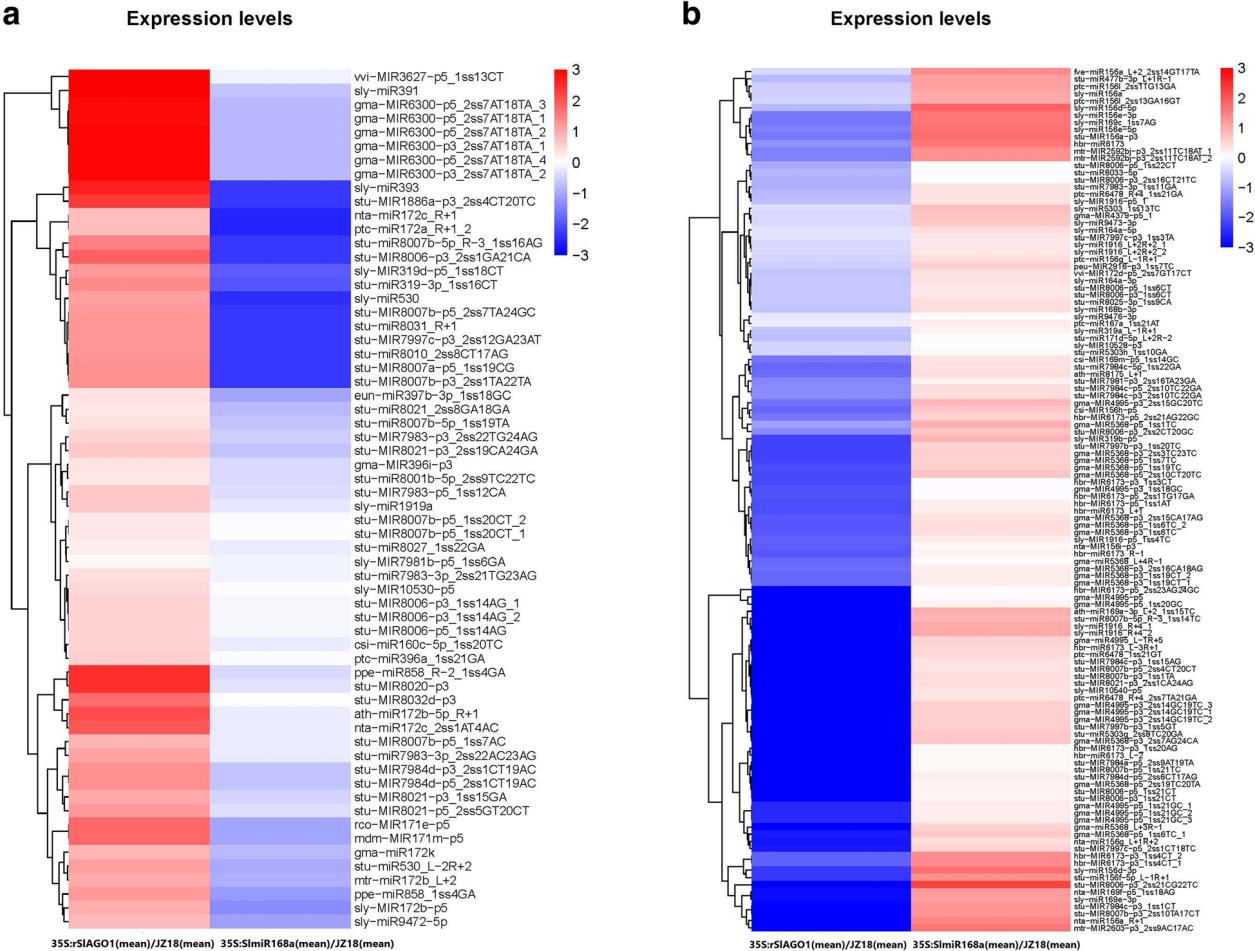
Heatmap showing differential expressed miRNAs. **a**. The differential expressed miRNAs that were significantly up-regulated in *35S:rSlAGO1* ‘Line 2’ compared with WT, but down-regulated in *35S:SlmiR168a* ‘Line 4’ compared with WT; **b**. The differential expressed miRNAs that were significantly down-regulated in *35S:rSlAGO1* ‘Line 2’ compared with WT, but up-regulated in *35S:SlmiR168a* ‘Line 4’ compared with WT. Color panels illustrate the log2 value of fold change

When comparing *35S:SlmiR168a* plants with WT plants, 122 miRNAs expression levels were significantly upregulated (fold change > 2; *P* < 0.1), whereas 110 miRNAs expression levels were significantly downregulated (fold change > 2; *P* < 0.1; Fig. 5b; Table S5). When comparing *35S:rSlAGO1* plants with WT plants, 102 miRNAs expression levels were significantly upregulated (fold change > 2; *P* < 0.1), whereas 58 miRNAs expression levels were significantly downregulated (fold change > 2; *P* < 0.1; Fig. 5b; Table S6). 62 known miRNAs which expression levels were upregulated when *35S:rSlAGO1* comparing to WT plants, but downregulated when *35S:SlmiR168a* comparing to WT plants were listed in Fig. 6a. There were 120 known miRNAs which expression levels were downregulated when comparing *35S:rSlAGO1* and WT plants, but upregulated when comparing *35S:SlmiR168a* and WT plants (Fig. 6b). The repression post-transcriptional regulation of the targets by the 62 known miRNAs might be induced by the RISC which containing AGO1 protein regulated by miR168 in *35S:rSlAGO1*.

### Functional analysis of miRNA predicted targets

**Fig. 7:**
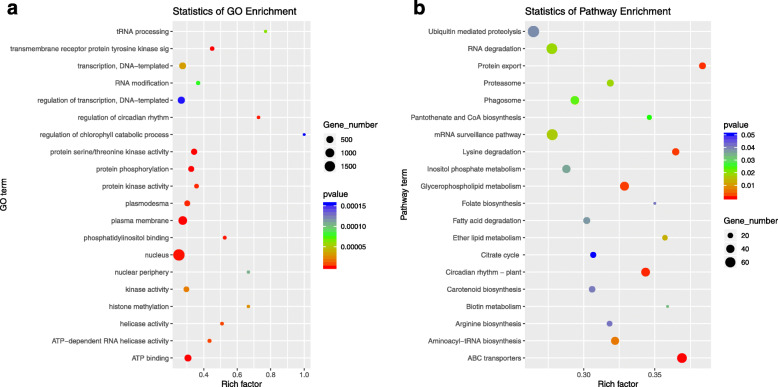
The predicted target genes of 107 differentially expressed miRNAs of both *35S:rSlAGO1* plants compared with WT and *35S:SlmiR168a* plants compared with WT. **a**. GO analysis of predicted targets of 107 differentially expressed miRNAs (20 terms). **b**. KEGG pathway enrichment analyses of predicted targets of 107 differentially expressed miRNAs (20 pathways)

One hundred seven differentially expressed miRNAs of both *35S:rSlAGO1* plants compared with WT and *35S:SlmiR168a* plants compared with WT were identified (Table S7), and the identified putative target genes were listed in Table S8. Gene ontology (GO) enrichment analysis for the predicted targets of the 107 miRNAs identified 20 terms, including nucleus, plasma membrane, and ATP binding, that changed significantly (*P* < 0.00015) between the two transgenic tomato plants compared with those in the WT plants (Fig. 7a). Pathway enrichment analysis for the predicted targets of the 107 miRNAs identified 20 pathways, including ABC transporters, glycerophospholipid metabolism, circadian rhythm-plant, and RNA degradation, that changed significantly (*P* < 0.05) between the two transgenic tomato plants compared with those in the WT plants (Fig. 7b).

### Integrated analysis of differentially expression miRNAs and mRNAs from *35S:SlmiR168a* and *35S:rSlAGO1* plants compared with WT plants

There is a regulatory relationship between miRNAs and mRNAs, and this relationship can be established through target gene prediction. In this study, we found 113 miRNA/mRNA predicted target pairs in the comparison of *35S:SlmiR168a* and WT plants, including positive and negative correlations (Table S9), and 93 miRNA/mRNA pairs in the comparison of *35S:rSlAGO1* and WT plants, including positive and negative correlations (Table S10). Owing to various regulatory factors, the expression of mRNAs by miRNAs did not have a completely inverse relationship, both positive and negative correlations were detected. In most cases that miRNAs promote the targets cleavage, the complementary expression pattern of miRNAs/mRNA pairs were chosen to be further analyzed. There were 74 negative miRNA/mRNA interaction pairs in the comparison of *35S:SlmiR168a* with WT (Table S9) and 49 negative miRNA/mRNA interaction pairs in the comparison of *35S:rSlAGO1* with WT (Table S10). Although AGO1 is known to be important for the stabilization of miRNAs, its role in miRNA production has not been established [27]. However, we chose 10 upregulated miRNA and downregulated mRNA interaction pairs in the comparison of *35S:rSlAGO1* with WT and two downregulated miRNA and upregulated mRNA interaction pairs in the comparison of *35S:SlmiR168a* with WT (Table 1). Thus, these miRNAs were thought to be stabilized by AGO1 protein. GO analysis of the 12 negative miRNA/mRNA pairs included 28 functional processes involving the CTK-activated signaling pathway, responses to salt stress, and responses to abscisic acid (ABA) (Figs. S1 and S3). Additionally, pathway enrichment analysis of the 12 negative miRNA/mRNA pairs included four pathways, involving plant/pathogen interactions, plant hormone signal transduction, base excision repair, and histidine metabolism (Fig. S2).

**Table 1:** Relative miRNA expression of 10 DE miRNAs for comparison of the*35S:SlmiR168a* versus WT groups and *35S:rSlAGO1* versus WT groups, in respect to by integrated analysis of mRNA-seq and miRNA-seq and Quantitative real-time PCR. * Asterisk indicates statistical significance of differential gene expression with *p*-value < 0.05 determined using a Duncan’s test compared with the WT. Inf, Infinite; FC, Fold Change; Sig FC, Significant Fold Change

miR_name	Compared group	FC	Sig FC	Regulation	RT-PCR	mRNA	FC	Sig FC	Regulation	RT-PCR
stu-miR530_L-2R + 2	35S:rSlAGO1/ JZ18	2.19	yes	up	1.96*	Solyc04g008110.3.1	0.20	yes	down	−3.78*
						Solyc07g063510.3.1	0.45	yes	down	−3.33*
ppe-miR858_1ss4GA	35S:rSlAGO1/ JZ18	2.33	yes	up	4.45*	Solyc05g006420.3.1	0.40	yes	down	−2.11*
ath-miR171a-3p_L-3R + 1	35S:rSlAGO1/ JZ18	inf	yes	up	inf*	Solyc08g069180.3.1	0.33	yes	down	−2.23*
stu-miR8039_R + 3_1ss4CT	35S:rSlAGO1/ JZ18	inf	yes	up	13.98*	Solyc12g056040.1.1	0.11	yes	down	−6.94*
stu-miR384-5p_R + 1	35S:rSlAGO1/ JZ18	inf	yes	up	10.45*	Solyc03g113890.1.1	0.09	yes	down	−9.65*
						Solyc06g076850.3.1	0.43	yes	down	−1.54*
PC-3p-276756_24	35S:rSlAGO1/ JZ18	inf	yes	up	2.23*	Solyc05g006420.3.1	0.40	yes	down	−2.02*
PC-5p-289257_23	35S:rSlAGO1/ JZ18	inf	yes	up	7.62*	Solyc04g082420.3.1	0.37	yes	down	−3.29*
PC-5p-66618_119	35S:rSlAGO1/ JZ18	4.29	yes	up	4.79*	Solyc08g066260.3.1	0.20	yes	down	−5.79*
stu-MIR8006-p3_1ss8GA_1	35S:SlmiR168a / JZ18	-inf	yes	down	-inf*	Solyc09g097780.2.1	2.59	yes	up	3.32*
stu-MIR8007b-p3_1ss22CT	35S:SlmiR168a / JZ18	0.48	yes	down	−1.98*	Solyc09g064820.1.1	6.60	yes	up	5.89*

### RT-PCR identification of differentially expressed miRNAs and mRNAs

The expression patterns of 10 differentially expressed known miRNAs (*stu-miR530_L-2R + 2*, *stu-miR-8039_R + 3_1ss4CT*, *stu-miR-384-5p_R + 1*, *ppe-miR-858_1ss4GA*, *ath-miR-171a-3p_L-3R + 1*, *PC-3p-276756_24*, *PC-5p-289257_23*, *PC-5p-66618_119*, *stu-miR-8006-p3_1ss8GA_1*, and *stu-miR-8007b-p3_1ss22CT*) and their 12 differentially expressed target genes (*Solyc04g008110.3.1*, *Solyc07g063510.3.1*, *Solyc03g113890.1.1*, *Solyc06g076850.3.1*, *Solyc05g006420.3.1*, *Solyc08g069180.3.1*, *Solyc12g056040.1.1*, *Solyc05g006420.3.1*, *Solyc04g082420.3.1*, *Solyc08g066260.3.1*, *Solyc09g097780.2.1*, and *Solyc09g064820.1.1*) were further performed by quantitative RT-PCR (qRT-PCR) (Table 1). These miRNAs/mRNAs pairs showed the same expression patterns as those performed in the miRNA-Seq/mRNA-Seq data. These similar expression tendencies suggested that the sequencing data were reliable for the further study.

### Correlation analysis of miRNAs and their target genes responsive to K^+^ deficiency stress

**Fig. 8:**
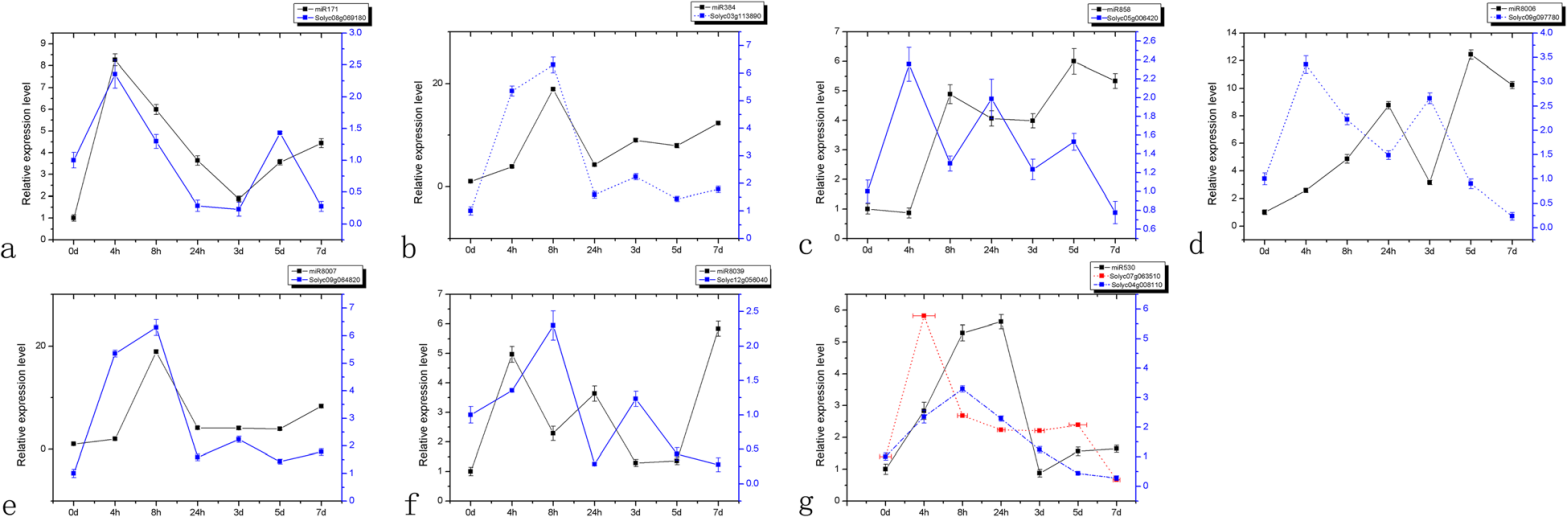
Quantitative real-time PCR validation of seven differentially expressed miRNAs and their predicted target genes in low K^+^ sensitive JZ18 tomatoes under normal conditions and K^+^ deficiency stress (0.5 mM) at the time 0d, 4 h, 8 h, 24 h, 3d, 5d and 7d. The experiments were repeated three times

From the 10 differentially expressed mature miRNAs, 7 known miRNAs and their 8 target genes were chosen. Then their expression under K^+^ deficiency stress was evaluated by RT-PCR (Fig. 8). Based on our results, 5 miRNA-target pairs (*stu-miR530/protein YnbB-like*, *stu-miR530/histidine kinase 4*, *stu-miR8039/endochitinase A-like*, *ppe-miR858/ARR5*, and *stu-miR8006/cold and drought-regulated protein CORA-like*) exhibited a negative relationship at the expression level and, indicating that a transcriptional repression may be mediated on these targets through corresponding miRNAs under K^+^ deficiency stress. Furthermore, three other miRNA-target pairs (*ath-miR171a/*U-box domain-containing protein 52-like, *stu-miR8007b*/EID1-like F-box protein 3, and stu-miR384/protein LOC107012202 isoform X1) demonstrated a similar expression tendency, although the expression pattern at 7 days after K^+^ deficiency stress was complementary. Importantly, the miRNAs expression levels were all up-regulated at 7 days after K^+^ deficiency stress, whereas expression levels of their targets were down-regulated. Thus, the expression levels of these miRNAs-target pairs indicated their response to K^+^ deficiency stress with the time earlier or later.

### CTK/ABA regulation by *SlmiR168* mediated *SlAGO1A* involved in the tolerance to K^+^ deficiency stress in tomato plants

**Fig. 9:**
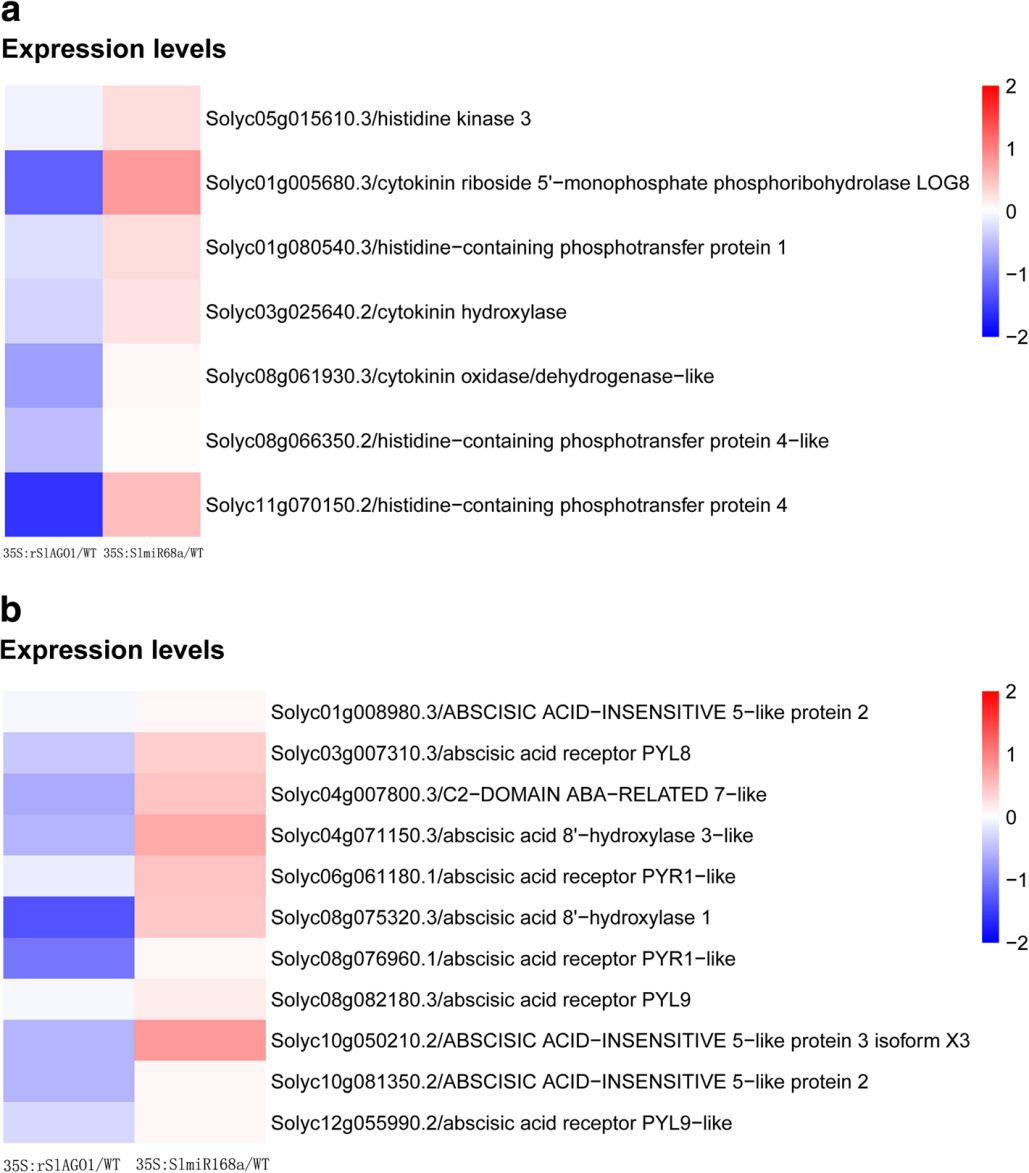
Heatmap showing DEGs encoding proteins related to abscisic acid (ABA) signalling and biosynthesis (**a**) and cytokinin (CTK) signalling and biosynthesis (**b**) in *35S:SlmiR168a* compared with WT and *35S:rSlAGO1* compared with WT respectively. Color panels illustrate the log2 value of fold change

**Fig. 10:**
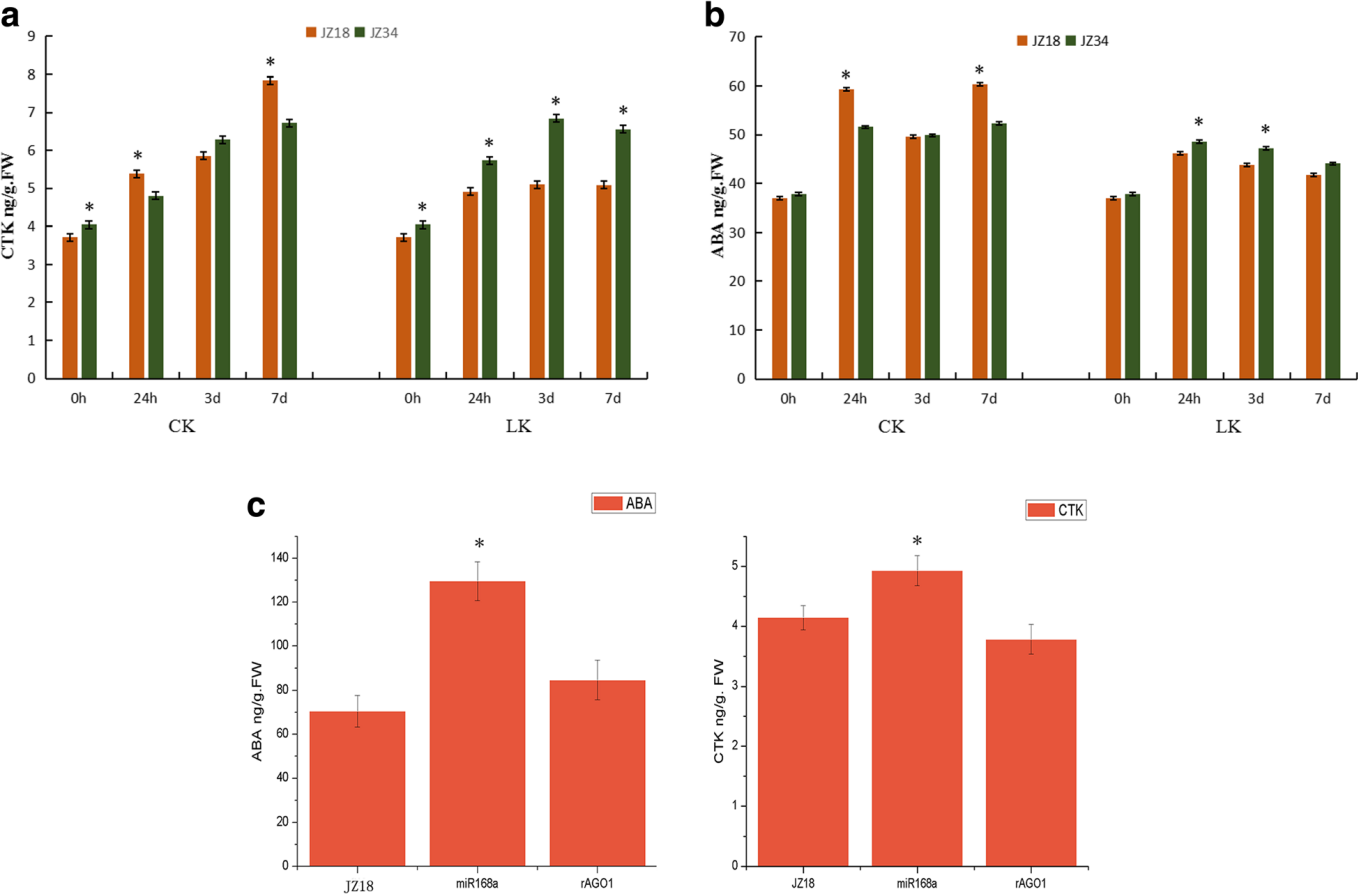
Comparison of CTK and ABA contents in low K^+^ sensitive (JZ18) and low K^+^ tolerant (JZ34) tomatoes under K^+^ deficiency stress conditions after 24 h, 3 days, and 7 days (**a** and **b**); CTK and ABA contents of JZ18, *35S:SlmiR168a* and *35S:rSlAGO1* under normal condition (**c**). CK: normal K^+^ (4 mM); LK: K^+^ deficiency (0.5 mM). The experiments were repeated three times. Error bars indicate the means ± SE of three independent replicates. * Significant differences with P < 0.05 determined using a Duncan’s test compared with the control

We found many genes involved in the biosynthesis and signaling of CTK and ABA biosynthesis that were downregulated in *35S:rSlAGO1* and upregulated in *35S:SlmiR168a* plants (Fig. 9a and b). This result prompted us to further investigate the influence of *SlmiR168*-mediated *SlAGO1A* regulation of the CTK and ABA pathway. Indeed, we found that the CTK and ABA contents in JZ18 and JZ34 tomatoes were different under K^+^ deficiency stress (Fig. 10). In particular, the JZ34 CTK content was significantly higher than that in JZ18 as the low-K^+^ treatment time increased (Fig. 10a). However, under normal conditions, the CTK content was lower in JZ34 plants than that in JZ18 plants. The same pattern was observed for the ABA content (Fig. 10b). The CTK/ABA contents were also investigated in *35S:SlmiR168a* and *35S:rSlAGO1*. The CTK and ABA content were significantly higher in *35S:SlmiR168a* than JZ18 and *35S:rSlAGO1* (Fig. 10c). Our results suggest that the CTK/ABA biosynthesis and signaling pathways were enhanced in *35S:SlmiR168a* which showed tolerance to low-K^+^ stress, but lowered in *35S:rSlAGO1* which showed sensitive to low-K^+^ stress.

## Discussion

**Fig. 11:**
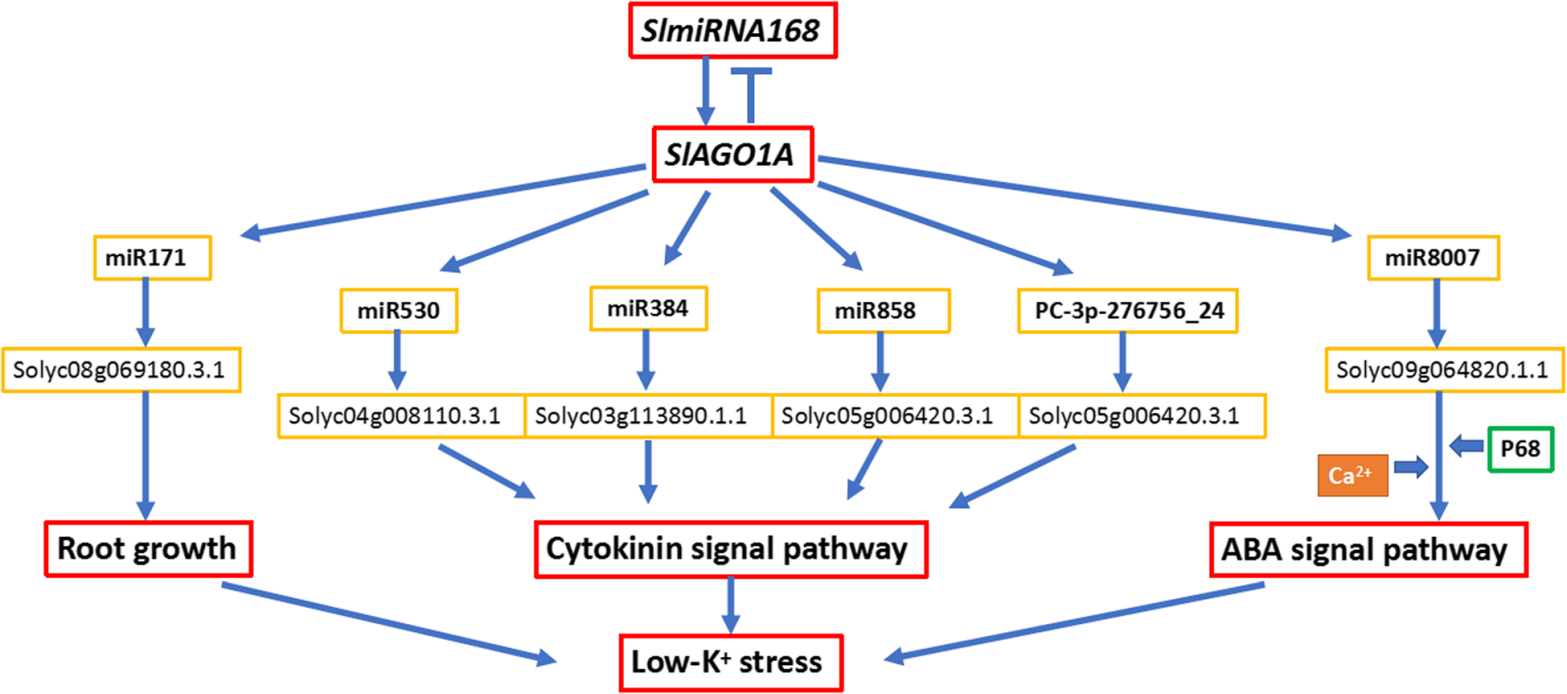
Hypothetical model of the *SlmiR168*-mediated *SlAGO1A* regulation upon K^+^ deficiency stress response. *SlAGO1A* regulation by *SlmiR168* is involved in various processes, including root growth, the CTK signalling pathway, and the ABA signalling pathway, by influencing the regulatory pathways of other miRNAs (e.g., ath-miR171a, stu-miR530, stu-miR384, ppe-miR858 and stu-miR8007b)

K^+^ deficiency in soil is of great agricultural importance [36]. One important aspect of plant adaptation to K^+^ deficiency stress is cellular and tissue homeostasis of K^+^, which involves transport of K^+^ across various membranes in several tissues [37]. The two tomato genotypes low-K^+^-tolerant JZ34 and low-K^+^-sensitive JZ18 exhibit marked differences in sensitivity to K^+^ deficiency and root morphology [9]. Moreover, JZ34 has more root hairs under K^+^ deficiency treatment than JZ18 and exhibits stronger nutritional uptake capability of K^+^ than JZ18 [9]. Thus, JZ34 maintains higher K^+^ contents under K^+^ deficiency stress than JZ18. Plants cannot escape from the various environmental stress, they have developed complex regulatory mechanisms in response to the effects of these stress [38]. For further exploring the molecular mechanisms of difference between JZ18 and JZ34, we found that the expression of *SlmiR168* was increased in response to low K^+^, whereas the expression of its target *SlAGO1A* was decreased following low K^+^ treatment in JZ34 (Fig. 1). Both *SlmiR168* and *SlAGO1A* were expressed at higher levels in roots than in other tissues (Fig. 2). Additionally, *35S:SlmiR168a* had more root hairs than *35S:rSlAGO1* and JZ18 (Fig. 4a). Actually, in addition to the roots, we also found difference in plant height between *35S:SlmiR168a and 35S:rSlAGO1* plants (Fig. S9). Notably, the potassium deficiency signal is first perceived by root cells, particularly root epidermal cells and root hair cells [7]. So the root phenotype seems more important in response to low K^+^ stress. *35S:SlmiR168a* showed more tolerant to low K^+^ deficiency than *35S:rSlAGO1.* Furthermore, the mRNA-Seq also demonstrated some Potassium transport genes were differetially expressed in *35S:SlmiR168a and 35S:rSlAGO1* (Table S11 and S12). Integrated analysis of mRNA-Seq and miRNA-Seq results in *35S:rSlAGO1* showed that a member of the *miR171* family was significantly induced and that its target *Solyc08g069180.3.1* was downregulated (Table 1). Further analysis showed that this target gene was involved in root epidermal cell differentiation and stress responses. Previous studies have shown that *miR171* expression is higher in the vascular bundle and cuticle layer of roots in *Arabidopsis* [39] and that this miRNA is upregulated in response to Cd stress, drought, and salt stress [40, 41]. *miR171* has also been shown to be differentially expressed in maize roots in response to salt stress [42]. miR171 was showed to be upregulated in 4 m-SlAGO1A plants compared to the wild type [26]. Therefore, we concluded that regulation of *Solyc08g069180.3.1* by *miR171a* may explain differences in root development between *35S:SlmiR168a* and *35S:rSlAGO1* under K^+^ deficiency stress (Fig. 11).

In addition to root architecture, phytohormones are also involved in signal transduction of plant responses to K^+^ deficiency stress. Low K^+^ stress results in decreased CTK levels, which may stimulate ROS accumulation, root hair growth, and *AtHAK5* expression [19]. The KAT1 potassium channel is a target for ABA signal transduction through SRK1/OST1/SnRK2.6 [43]. Additionally, expression of the K^+^ release channel gene *GORK* is induced by ABA in the presence of extracellular Ca^2+^ [44]. In this study, we found that the low K^+^ tolerant tomato JZ34 had higher CTK/ABA contents under K^+^ deficiency stress than the low K^+^ sensitive tomato JZ18. Integrated analysis of mRNA-Seq and miRNA-Seq results for the comparison of *35S:rSlAGO1* versus JZ18 showed that *miR384*, *miR530*, and *miR858* were upregulated and that their downregulated targets were enriched in the CTK signaling pathway and CTK responses. CTK accumulation decreases ROS production [19]. The excessive production of ROS that damage macromolecules, including lipids, proteins and so on in plant cells [45]. So JZ34 was more tolerant to K^+^ deficiency stress than JZ18, probably causing by the SlAGO1A regulated by miR168 to influence CTK signal adjusting the damage of the ROS on the plant cells. Moreover, we found that targets of *miR384*, *miR530*, and *miR858* were also involved in plant hormone signal transduction by KEGG analysis. Additionally, target genes of the novel miRNA *PC-3p-276756_24* were found to be involved in CTK responses. Interestingly, in *35S:SlmiR168a*, only *miR8006* and *miR8007b* were downregulated, and their upregulated targets were upregulated and enriched in response to salt stress and ABA. Accordingly, our results showed that *SlAGO1A* induced repression of the targets expression which regulated by various miRNAs, including *miR384*, *miR530*, *miR858*, *miR8007*, and *PC-3p-276756_24*, through the regulation of *SlmiR168a*. What is more, the CTK/ABA content were especially increased in *35S:miR168a* in our study. These miRNA/mRNA pairs may influence tolerance to K^+^ deficiency stress in plants via the CTK/ABA signaling pathway (Fig. 11). K^+^ transport via ABA signaling requires extracellular Ca^2+^ [44], and P68 protein combines with AGO1 to interact with CaM and enhance accumulation of K^+^ in rice [46]. Thus, P68 expression was investigated in *35S:SlmiR168a* and *35S:rSlAGO1* (Fig. S4). P68 expression levels were decreased in *35S:rSlAGO1* but increased in *35S:SlmiR168a* compared with those in WT. Based on these findings, the pathway through which *SlAGO1A* was regulated by *SlmiR168* in response to K^+^ deficiency stress via ABA signaling may require Ca^2+^.

miRNAs are loaded onto *AGO1*, which acts as an RNA slicer in plants [47]. *miR168* directs the cleavage of *AGO1* mRNA, indicating that *miR168* regulates the activity of its own miRNA pathway [33]. *AGO1*-null alleles reduce the expression levels of some miRNAs, such as *miR171*, and increase the levels of the corresponding target mRNAs [33]. In *35S:SlmiR168a*, the expression of miR168 indicates the following regulation pathway: *SlmiR168* is up-regulated, the accumulation of *SlmiR168* represses the expression of *SlAGO1A*. As losing the *SlAGO1A* binding to the RISC, the expression of the targets regulated by other miRNAs which bind to the RISC containing *SlAGO1A* would be up-regulated. So we tend to discover the expression of targets is up-regulated by the miRNAs expression which were down-regulated in *35S: SlmiR168a*. Moreover, in *35S:SlmiR168a*, miR8006 and miR8007 were downregulated, and their targets were upregulated. In *35S:rSlAGO1*, the expression of *rSlAGO1A* is up-regulated, and *rSlAGO1A* cannot be regulated by *SlmiR168,* so the function of RISC is promoted to bind the downstream miRNAs. So the targets post-transcriptional regulation of other miRNAs which binding to RISC containing *SlAGO1A* would be enhanced. Finally, we found eight miRNAs were upregulated, and their targets were downregulated in *35S:rSlAGO1*. Thus, these miRNAs above may be associated with AGO1A protein, and impairment of AGO1-miR168 feed-back regulation could disturb the maintenance of suitable *SlAGO1A* for the plant development and response to the environment. Additionally, in *35S:SlmiR168a*, 71 miRNAs were also found to be upregulated, and their targets were downregulated. These miRNAs included many widely known molecules, such as *miR167*, *miR156*, *miR396*, *miR166*, *miR319b*, and *miR172*. These miRNAs may be involved in various hormone signaling pathways, including auxin, ethylene, and gibberellin signaling. In *AGO1-*null plants, *miR156*/*157* and *miR167* were also found to accumulate to levels similar to or higher than those in WT plants [33]. Furthermore, Lynn et al. [48] reported that PINHEAD/ZWILLE is 75% similar to and has overlapping functions with AGO1. AGO proteins can bind to single-stranded RNAs that are at least 5 nt in length and to double-stranded RNA, enabling AGO protein to directly associate with miRNAs before and after they recognize their mRNA targets [49]. AGO1 might even act before miRNA processing [32]. The tomato AGO family have 15 members, and besides *SlAGO1A* and *SlAGO1B* were regulated by *SlmiR168a*, the *SlAGO2A* was also regulated by miR403 [30]. If the overaccumulation of *SlmiR168* influences other AGO family members or other miRNAs needs further confirmation. In addition, our results showed that *SlmiR168*-targeted *SlAGO1A* may be involved in K^+^ deficiency stress in shoot and root. *35S:SlmiR168a* plants displayed more tolerance to K^+^ deficiency stress both in root and shoot compared to WT plants under K^+^ deficiency stress (Fig. 4a and b). However, *35S:rSlAGO1* plants could show more sensitive to K^+^ deficiency stress in shoot than in root (Fig. 4b). Homology of *SlAGO1A* and *SlAGO1B* was 88%, but the percentage of Q in *SlAGO1B* was much higher than that in *SlAGO1A* [30]. The expression of *SlAGO1A* was different from that of *SlAGO1B* in fruit development [30]. 4 m-SlAGO1A demonstrated a little different defects in flowers from the 4 m-SlAGO1B transformants [26]. These indicates that *SlAGO1A* and *SlAGO1B* might play different roles in tomato development. Under K^+^ deficiency stress, the other SlAGO1 protein, SlAGO1B may mainly cause the difference in root phenotype between *35S:SlmiR168a and 35S:rSlAGO1* plants. Importantly, miRNAs are regulated by AGO in time and space, resulting in finely-tuned and complex regulatory networks. Thus, miR168 may function with AGO1 to control the mRNA levels of miRNA targets through a complex network.

Based on analysis of miRNAs and mRNAs responding to K^+^ deficiency stress, we developed a model of miR168-mediated AGO1 function in low K^+^ tolerance (Fig. 11). *SlAGO1A* is regulated by *SlmiR168* in response to K^+^ deficiency stress, and overexpression of *SlAGO1A* then induces the expression of *miR530*, *miR384* and *miR858*, resulting in enhancing the post-transcriptional silencing of the targets regulated by these miRNAs, which participate in CTK signaling. *SlAGO1A* accumulation also induces *miR171* expression and then downregulates its targets, which are involved in root epidermal cell differentiation to inhibit the root hair growth under low-K^+^ stress. Moreover, *SlmiR168*-mediated *SlAGO1A* regulates the expression of *miR8007*, which is involved in the ABA signaling pathway; Ca^2+^ may have functions in this pathway as well. So the regulation of *SlmiR168* on the *SlAGO1A* is vital for the maintaining *SlAGO1* at a steady level to maintain the normal plant growth under low-K^+^ stress.

## Conclusion

In this study, the RISC containing *SlAGO1A* regulated by *SlmiR168* influenced part of the other miRNAs post-transcriptional regulation. These miRNAs (*miR530*, *miR384*, *miR858*, miR171 and miR8007) further target various mRNA in response to low-K^+^ in different pathways by modulation of root growth and CTK/ABA biosynthesis and signaling. The overexpression of *pri-SlmiR168a* improves the tolerance of tomato plants in response to low-K^+^ stress. Collectively, our results revealed new regulation pathways of *SlmiR168*-mediated *SlAGO1A* in response to low K^+^ stress and highlighted the importance of *SlAGO1A* in maintaining the homeostasis of miRNA accumulation. This study provides new perspectives in the molecular and breeding mechanisms to improve the tolerance of tomato plants to low-K^+^ environmental stress.

## Methods

### Plant materials and growth conditions

Two tomato genotypes ‘JZ34’ (low K^+^-tolerant) and ‘JZ18’ (low K^+^-sensitive) were obtained in our lab by higher generation inbred lines and introduced in detail about the low-K^+^ tolerance by Zhao et al. in 2018 [9]. These tomato seeds were saved in our lab. These tomato seedlings were grown under standard greenhouse conditions, including a day/night temperature condition of 26/18 °C with a photoperiod of 16 h light/8 h dark. 25 days old seedlings were washed with water, and transferred to pot for nutrient hydroponics. The nutrient solution formula was performed as described previously [9]. At the vegetative growing stage (30 days), a K^+^ − deficient condition was induced by reducing the concentration of KNO_3_ from 4 mM (normal K^+^) to 0.5 mM (K^+^ deficiency) in the nutrient solution. Nutrient solution with 4 mM KNO_3_ was used as the control. After 7 days of K^+^ deficiency stress, different parts of the plant were sampled to assess plant root configuration, fresh weight, and K^+^ content.

### Measurement of K^+^ concentrations

A total of 0.05 g (dry weight) tomato roots were added to a 10-mL centrifuge tube containing 2 mL of 0.5 M hydrochloric acid. Samples were incubated for 3 days, after which, 5 mL deionized water was added to each centrifuge tube, and the mixture was filtered. The filtered stock solution was diluted 10 times, and the K^+^ concentration was measured with a flame photometer. Each sample was evaluated with three biological replicates.

### *35S:SlmiR168a* and *35S:rSlAGO1* vector construction and tomato transformation

*Pri-SlmiR168a* was prepared using gene-specific primers. The sequence-confirmed polymerase chain reaction (PCR) fragment was cloned into the pCAMBIA3301/Luc plasmid, which contained two 35S Cauliflower mosaic virus promoters, the marker gene for kanamycin resistance, phosphinothricin, and luciferase. Recombinant plasmids containing the expected insert were transferred into *Agrobacterium tumefaciens* GV3101 cells. The competent cells harboring the vector were transformed into JZ18 tomatoes using a tomato genetic transformation system [50]. Expression of the target gene was detected in the T1 transformants and their corresponding T2 using qRT-PCR along with detection of the presence of the kanamycin marker gene. All primers used in this study are listed in Supplementary Table S1. To generate *rSlAGO1A* (the *SlmiR168*-resistant construct), mutations in the *SlmiR168* target site of *SlAGO1A* were inserted using two-step PCR mutagenesis. The *35S:SlAGO1* transformants were obtained using the same method described above for *35S:SlmiR168a*.

### Small RNA sequencing and analysis of differentially expressed miRNAs

*35S:SlmiR168a*, *35S:rSlAGO1*, and JZ18 were used as small RNAs. In total, nine samples (*35S:SlmiR168a*, *35S:rSlAGO1*, and JZ18, each with three replicates) were harvested. About 2.5 μg total RNA obtained from the tomato leaves was used to construct small RNA library by TruSeq Small RNA Sample Prep Kits (Illumina, San Diego, CA, USA). Then sequencing was used by an Illumina Hiseq2500 50SE platform (single end) at LC-BIO (Hangzhou, China) following the manufacturer’s instructions. The detailed information of sequencing was referred to description previously reported [51]. The identification of conserved and novel miRNAs are summarized in Table S2.

### Prediction of target genes of miRNAs

GSTAr.pl was used to predict the genes targeted by the differentially expressed miRNAs. The minimum free energy (MFE) of miRNA-cDNA duplexes was calculated with the RNAhybrid program [52–54] with the following parameters: MFE ratio ≥ 0.65; and Allen Score ≤ 10. Then, a modified version the CleaveLand4 program was used to identify the potential cleavage sites of miRNAs in the corresponding targets based on degradome data http://sites.psu.edu/axtell/software/cleaveland4/) [55].

### RNA exaction and transcriptome sequencing, annotation

Tomato leaflet samples were collected from JZ18, *35S:rSlAGO1*, and *35S:SlmiR168a* plants at the same stage and position, total RNA was extracted and Illumina Miseq libraries were constructed, following the manufacturer’s instructions. Each sample had three biological replicates. The mRNA which were used polyT oligos magnetic beads was purified from the total RNA. The fragments were cleaved by the fragmentation buffer. The first-strand cDNA was synthesized by using random hexamer primers and then transformed into double-stranded cDNA using RNase H and DNA polymerase I, and then linked with sequencing adapters. The sequencing library was constructed by PCR amplification and performed by using the Illumina Hiseq 2500 platform (LC-BIO Technology Co., Ltd.). For functional annotation, the differential expressed genes enrichment analyses were performed by Gene Ontology (GO) Blast2GO software (http://www.blast2go.org/) and KEGG Automatic Annotation Sever (http://www.genome.jp/tools/kaas/).

### Integrated analysis of mRNA-seq and miRNA-seq data

CGT101-CORR 1.1 software was used to define the possible positive and negative interactions between miRNA and mRNA were used A According to the constructed miRNA/mRNA regulatory network, the integrated analysis of miRNA-seq with mRNA-seq data was performed by combining the differentially expressed miRNAs and mRNAs with the associated miRNA-targeting information. Then the differentially expressed miRNA-targeting information was also taken into account.

### qRT-PCR analysis

Total RNA from the samples of leaves was extracted using TRIzol (Takara, Dalian, China) followed by RQ1 Dnase I (Promega, Madison, WI, USA) treatment to remove genomic DNA contamination. DNA-free RNA (2 μg) was used for cDNA synthesis. For the mature miRNA expression detection is used by the RT primer, which effectively binds to the 3′ end of the miRNA. The RT-PCR for target mRNAs and mature miRNA system was performed as described previously [56]. The templates were mixed with the SYBR Green PCR Master Mix on the ABI 7500 sequence detection system and software (Applied Biosystems, USA). Each measurement was repeated using three technical replicates, and the RNA samples of three biological replicates were mixed. The expression levels were normalized to the tomato U6 small nuclear RNA for miRNA quantification and actin was used for the mRNA quantification [56]. The primers are listed in Supplementary Table S1.

### ABA and CTK quantification

ABA and CTK were quantified using the enzyme-linked immunosorbent assay protocol [57]. The fresh tomato leaves were collected as weigh as 0.5 g–1.0 g. The method in detail for quantification of ABA and CTK was referred to the performance previously reported [56]. The absorbance of the antibodies against ABA and CTK was recorded at 490 nm. The samples of leaves were repeated with three biological replicates, and three technical replicates.

### Statistical analysis

At least three biological replicates were evaluated for all experiments; data are presented as the mean ± standard deviation. Statistical analyses (One-Way ANOVA by Duncan’s method) were performed using the SPSS software (version 17.0). A *P* < 0.05 was considered as statistically significant.

## Supplementary information

**Additional file 1:** **Table S1.** Primers used in this study.

**Additional file 2: Table S2.** The profiles of small RNA deep sequencing for *35S:SlmiR168a*, *35S:rSlAGO*1 and WT.

**Additional file 3: Table S3.** List of all expressed miRNA in *35S:SlmiR168a*, *35S:rSlAGO1* and WT.

**Additional file 4: Table S4.** The expressed conserved miRNAs were classified into different miRNAs families.

**Additional file 5: Table S5.** List of the differentially expressed miRNAs in *35S:SlmiR168a* plants compared with WT.

**Additional file 6: Table S6.** List of the differentially expressed miRNAs in *35S:rSlAGO1* plants compared with WT.

**Additional file 7: Table S7.** List of the miRNAs whose target genes are predicted.

**Additional file 8: Table S8.** Target predict annotation for the differentially expressed miRNAs.

**Additional file 9: Table S9.** miRNA/mRNA pairs in the comparison of *35S:SlmiR168a* and WT plants, with upregulated/upregulated,downregulated/downregulated, upregulated/downregulated, downregulated/upregulated by integrated analysis of miRNA-Seq and mRNA-Seq.

**Additional file 10: Table S10.** miRNA/mRNA pairs in the comparison of *35S:rSlAGO1* and WT plants, with upregulated/upregulated,downregulated/downregulated, upregulated/downregulated, downregulated/upregulated by integrated analysis of miRNA-Seq and mRNA-Seq.

**Additional file 11: Table S11.** List of the differentially expressed mRNAs in *35S:SlmiR168a* plants compared with WT.

**Additional file 12: Table S12.** List of the differentially expressed mRNAs in *35S:rSlAGO1* plants compared with WT.

**Additional file 13: Table S13.** The biomass of the roots and shoots in JZ18, *35S:miR168a* and *35S:rSlAGO1* under the normal and K^+^ deficiency stress conditions.

**Additional file 14: Figure S1.** GO analyses of the 10 negative miRNA/mRNA pairs identified in the comparison of *35S:rSlAGO1* and WT plants by integrated analysis of miRNA-Seq and mRNA-Seq.

**Additional file 15: Figure S2.** KEGG pathway enrichment analyses of the 10 negative miRNA/mRNA pairs identified in the comparison of *35S:rSlAGO1* and WT plants by integrated analysis of miRNA-Seq and mRNA-Seq.

**Additional file 16: Figure S3.** GO analyses of the 2 negative miRNA/mRNA pairs identified in the comparison of *35S:SlmiR168a* and WT plants by integrated analysis of miRNA-Seq and mRNA-Seq.

**Additional file 17: Figure S4.** Quantitative real-time PCR validation of P68 in *35S:SlmiR168a*, *35S:rSlAGO* and WT. The experiments were repeated three times.

**Additional file 18: Figure S5.** Comparison of morphological changes of root growth in WT, *35S:SlmiR168a*, and *35S:rSlAGO1* plants under normal K^+^ conditions and K^+^ deficiency stress after 7 days.

**Additional file 19: Figure S6.** Full-length *rSlAGO1* gel and blots. The red line inner part is the cropping part in Fig. 3b. The marker is 10,000 bp.

**Additional file 20: Figure S7.** Full-length of pre-SlmiR168a gel and blots. The black line inner part is the cropping part in Fig. 3c. The marker is 2000 bp.

**Additional file 21: Figure S8.** The expression levels of SlmiR168 in *35S:SlmiR168a* transformation lines; the expression levels of *SlAGO1A* in *35S:rSlAGO1* transformation lines. * Significant differences with *P* < 0.05 determined using a Duncan’s test compared with the WT.

**Additional file 22: Figure S9.** The phenotype of the whole plants of JZ18, *35S:SlmiR168a* and *35S:rSlAGO1* under the normal condition.

## Data Availability

The raw reads of this study were deposited in the SRA database (http://www.ncbi.nlm.nih.gov/sra/) at NCBI with SRA accession number (Small RNA sequencing: PRJNA615321; Transcriptome sequencing: PRJNA615315 and PRJNA615767).

## Abbreviations

ABA: Abscisic acid
CTK: Cytokinin
CK: Control check
LK: Low potassium stress
GO: Gene ontology
KEGG: Kyoto encyclopedia of genes and genomes
ABC transporter: ATP-binding cassette transporter
RISC: RNA induced silencing complex
RT-PCR: Reverse transcription polymerase chain reaction
WT: Wild type
ROS: Reactive oxygen species
CaM: Calmodulin
