# Correction to: miR168 targets Argonaute1A mediated miRNAs regulation pathways in response to potassium deficiency stress in tomato

**DOI:** 10.1186/s12870-021-02855-4

**Published:** 2021-02-08

**Authors:** Xin Liu, Chunchang Tan, Xin Cheng, Xiaoming Zhao, Tianlai Li, Jing Jiang

**Affiliations:** 1 Horticulture Department, College of Horticulture, Shenyang Agricultural University, No. 120 Dongling Road, Shenhe District, Shenyang, 110866 P. R. China; 2 Key Laboratory of Protected Horticulture of Ministry of Education, No. 120 Dongling Road, Shenhe District, Shenyang, 110866 P. R. China; 3 Key Laboratory of Protected Horticulture of Liaoning Province, No. 120 Dongling Road, Shenhe District, Shenyang, 110866 P. R. China

**Correction to: BMC Plant Biol 20, 477 (2020)**

**https://doi.org/10.1186/s12870-020-02660-5**

In the original publication [1] there was an incorrect reference citation. In this correction article the incorrect and correct information are listed.

**Incorrect**

- K^+^ deficiency in soil is of great agricultural importance [2].

**Correct**

- K^+^ deficiency in soil is of great agricultural importance.
