# Supplementary material for: miR168 targets Argonaute1A mediated miRNAs regulation pathways in response to potassium deficiency stress in tomato
Source: BMC Plant Biol. 2020 Oct 19;20:477. doi: 10.1186/s12870-020-02660-5 (PMC7574427; doi:10.1186/s12870-020-02660-5)
Supplement: Supplementary file 2 — Additional file 2: Table S2. The profiles of small RNA deep sequencing for 35S:SlmiR168a, 35S:rSlAGO1 and WT. [file 12870_2020_2660_MOESM2_ESM.doc]

**Table S2** The profiles of small RNA deep sequencing for *35S:SlmiR168a*, *35S:rSlAGO1* and WT.

| Smaple |  | Raw reads | Cut adapter and length filter | Junk reads | Rfam | mRNA | Repeats | Valid reads |
| --- | --- | --- | --- | --- | --- | --- | --- | --- |
| JZ18_1 | Total | 12836013 | 3773108 | 50593 | 476756 | 1519423 | 7522 | 7163035 |
|  | Unique | 3824961 | 1177977 | 31937 | 12493 | 31236 | 177 | 2575545 |
| JZ18_2 | Total | 14373027 | 1843786 | 101587 | 193627 | 1065934 | 3321 | 11223930 |
|  | Unique | 6007826 | 1212736 | 66679 | 7083 | 29045 | 175 | 4694297 |
| JZ18_3 | Total | 13912496 | 2960982 | 104579 | 181083 | 869285 | 3064 | 9849836 |
|  | Unique | 5879267 | 1365661 | 70323 | 7633 | 27973 | 153 | 4410072 |
| 35S:SlmiR168a | Total | 14850199 | 4270693 | 69711 | 469553 | 1678585 | 7868 | 8542869 |
|  | Unique | 4367984 | 1124988 | 42543 | 11930 | 32700 | 193 | 3159839 |
| 35S:SlmiR168a | Total | 17821390 | 4710630 | 86544 | 486224 | 1998416 | 7941 | 10694993 |
|  | Unique | 5163649 | 1375376 | 51838 | 11571 | 37366 | 200 | 3691188 |
| 35S:SlmiR168a | Total | 12006556 | 2656495 | 59563 | 360703 | 1485095 | 6508 | 7571073 |
|  | Unique | 4047201 | 1077134 | 37370 | 10521 | 29928 | 186 | 2895817 |
| 35S:rSlAGO1 | Total | 17470288 | 4442215 | 83238 | 373897 | 1992983 | 5336 | 10723320 |
|  | Unique | 5501369 | 1544215 | 51851 | 9722 | 35793 | 205 | 3862724 |
| 35S:rSlAGO1 | Total | 12383616 | 1946715 | 85095 | 173251 | 927488 | 3263 | 9305655 |
|  | Unique | 5240564 | 952187 | 58153 | 6976 | 25744 | 168 | 4199533 |
| 35S:rSlAGO1 | Total | 25030158 | 5536936 | 113940 | 678020 | 2213646 | 8696 | 16653370 |
|  | Unique | 7507525 | 1984484 | 68719 | 17836 | 47166 | 321 | 5393163 |
