# Supplementary figures and images for: miR168 targets Argonaute1A mediated miRNAs regulation pathways in response to potassium deficiency stress in tomato
Source: BMC Plant Biol. 2020 Oct 19;20:477. doi: 10.1186/s12870-020-02660-5 (PMC7574427; doi:10.1186/s12870-020-02660-5)

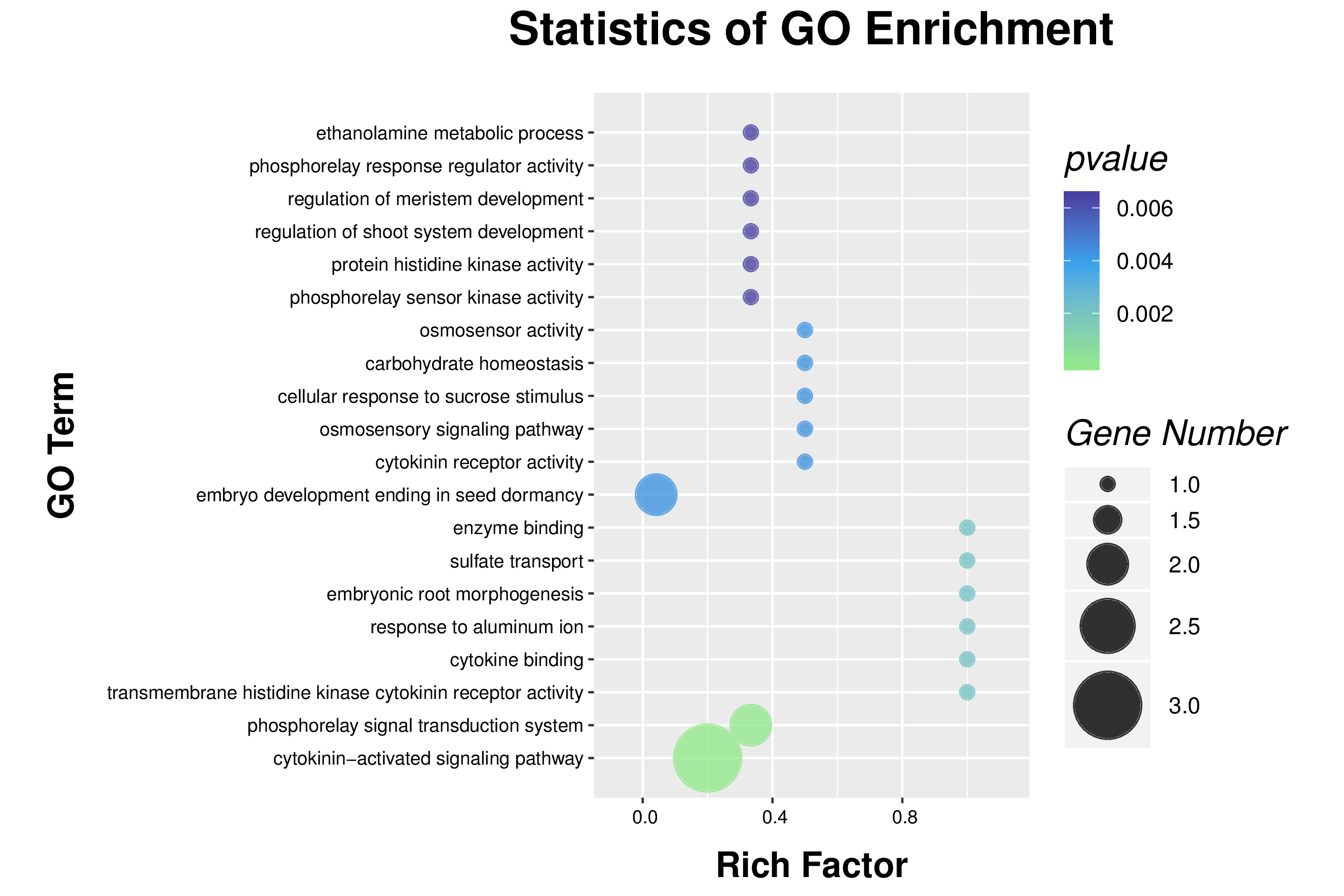

Supplement: Supplementary file 14 — Additional file 14: Figure S1. GO analyses of the 10 negative miRNA/mRNA pairs identified in the comparison of 35S:rSlAGO1 and WT plants by integrated analysis of miRNA-Seq and mRNA-Seq. [file 12870_2020_2660_MOESM14_ESM.tif]

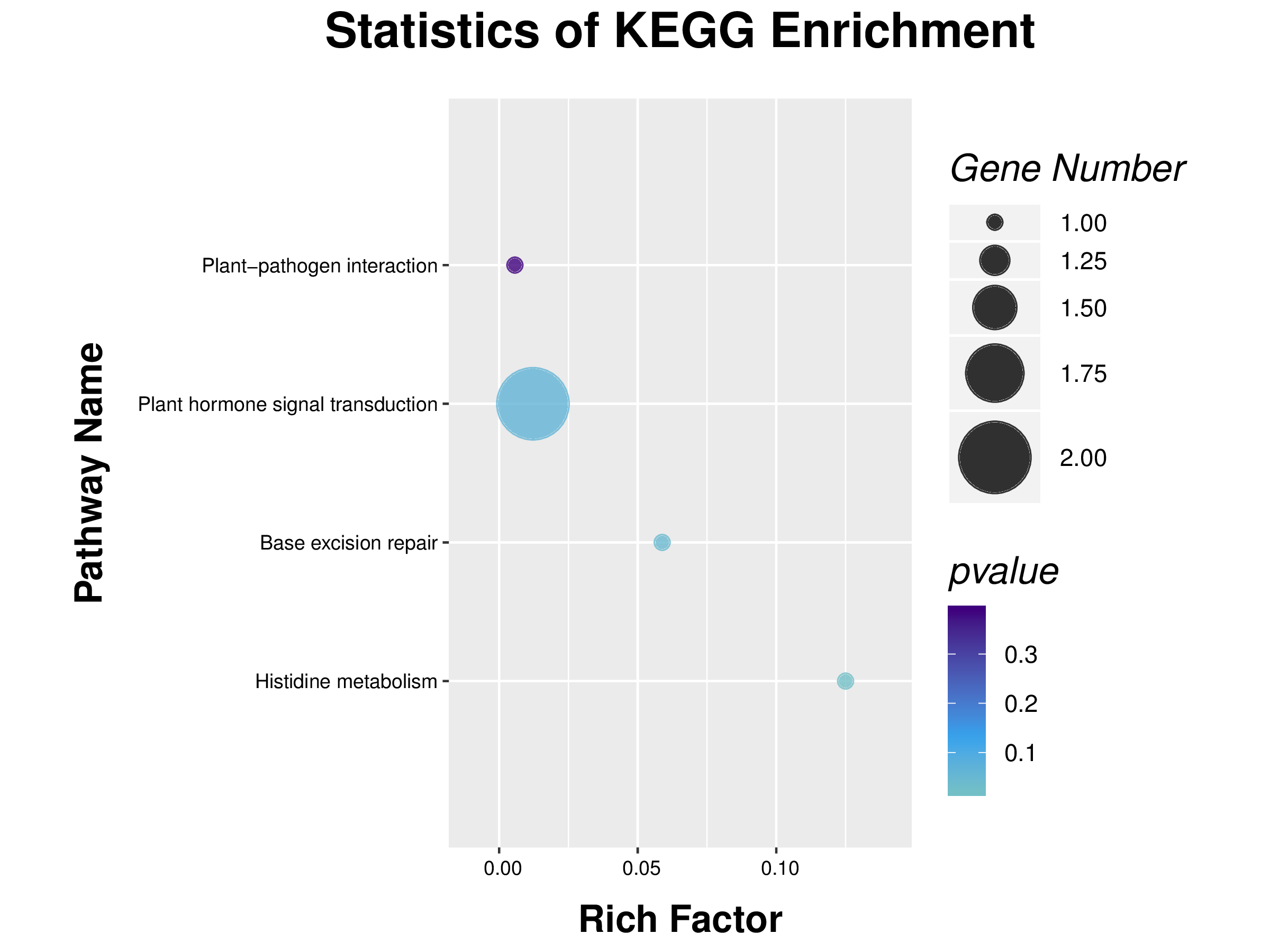

Supplement: Supplementary file 15 — Additional file 15: Figure S2. KEGG pathway enrichment analyses of the 10 negative miRNA/mRNA pairs identified in the comparison of 35S:rSlAGO1 and WT plants by integrated analysis of miRNA-Seq and mRNA-Seq. [file 12870_2020_2660_MOESM15_ESM.tif]

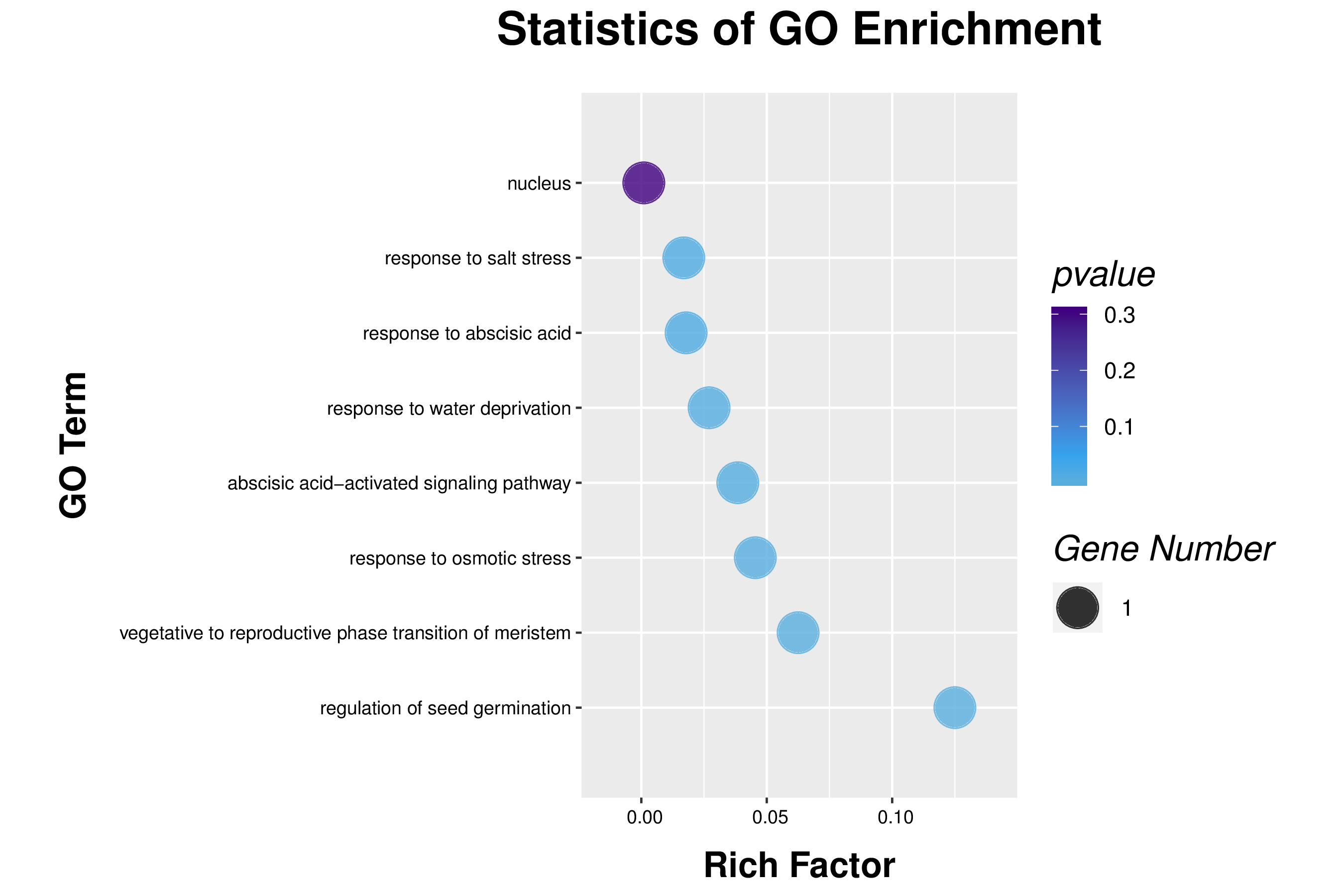

Supplement: Supplementary file 16 — Additional file 16: Figure S3. GO analyses of the 2 negative miRNA/mRNA pairs identified in the comparison of 35S:SlmiR168a and WT plants by integrated analysis of miRNA-Seq and mRNA-Seq. [file 12870_2020_2660_MOESM16_ESM.tif]

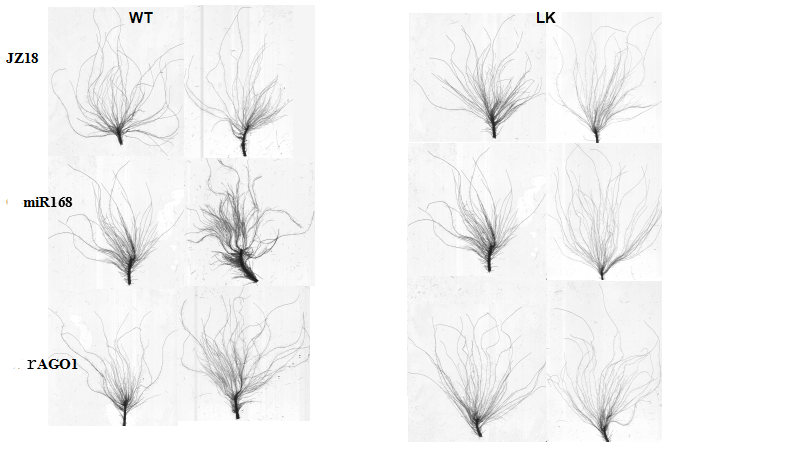

Supplement: Supplementary file 18 — Additional file 18: Figure S5. Comparison of morphological changes of root growth in WT, 35S:SlmiR168a, and 35S:rSlAGO1 plants under normal K+ conditions and K+ deficiency stress after 7 days. [file 12870_2020_2660_MOESM18_ESM.tif]

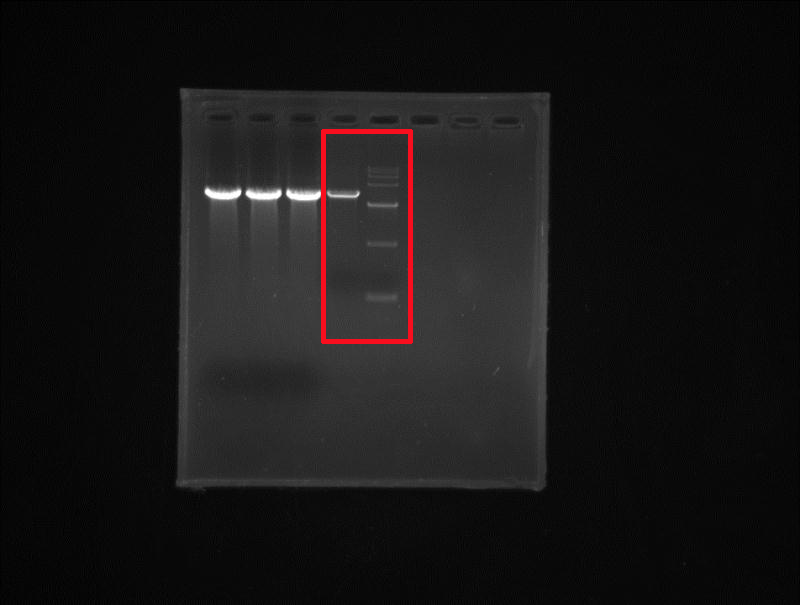

Supplement: Supplementary file 19 — Additional file 19: Figure S6. Full-length rSlAGO1 gel and blots. The red line inner part is the cropping part in Fig. 3b. The marker is 10,000 bp. [file 12870_2020_2660_MOESM19_ESM.tif]

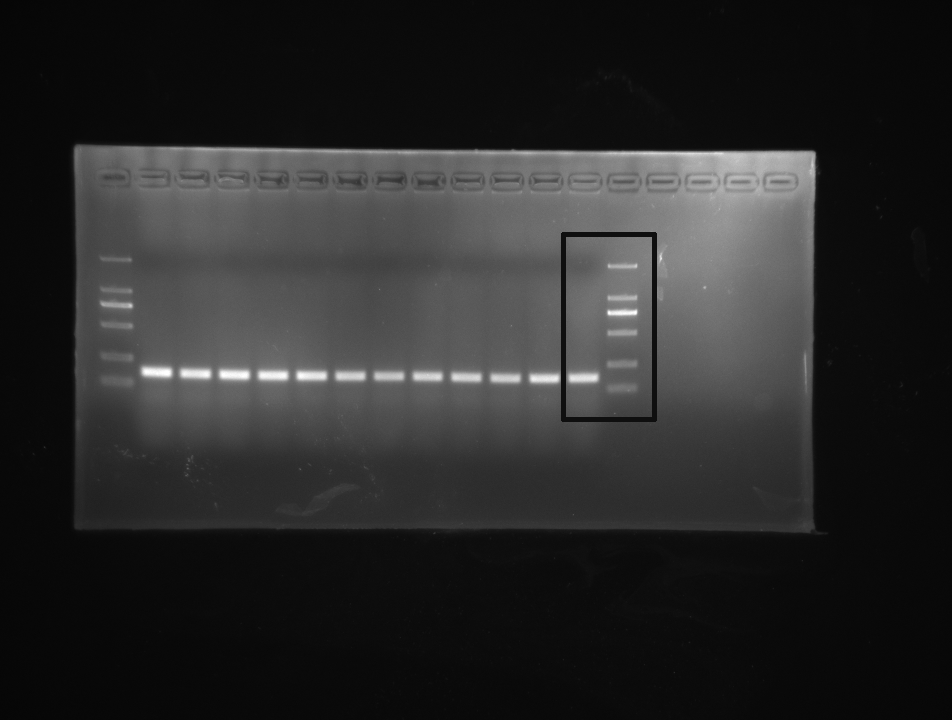

Supplement: Supplementary file 20 — Additional file 20: Figure S7. Full-length of pre-SlmiR168a gel and blots. The black line inner part is the cropping part in Fig. 3c. The marker is 2000 bp. [file 12870_2020_2660_MOESM20_ESM.tif]

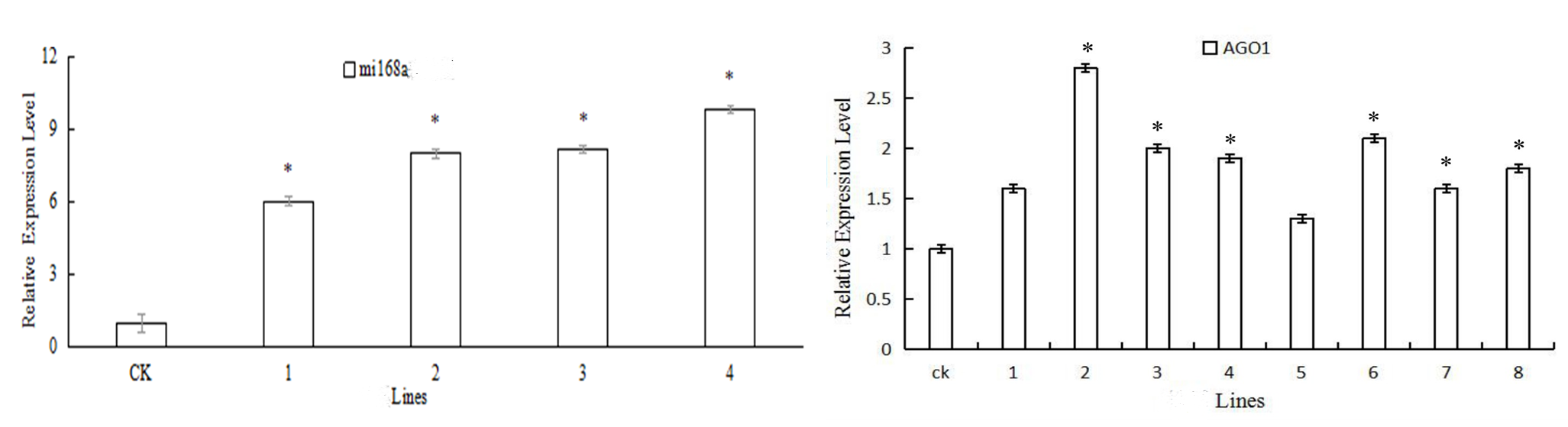

Supplement: Supplementary file 21 — Additional file 21: Figure S8. The expression levels of SlmiR168 in 35S:SlmiR168a transformation lines; the expression levels of SlAGO1A in 35S:rSlAGO1 transformation lines. * Significant differences with P < 0.05 determined using a Duncan’s test compared with the WT. [file 12870_2020_2660_MOESM21_ESM.tif]

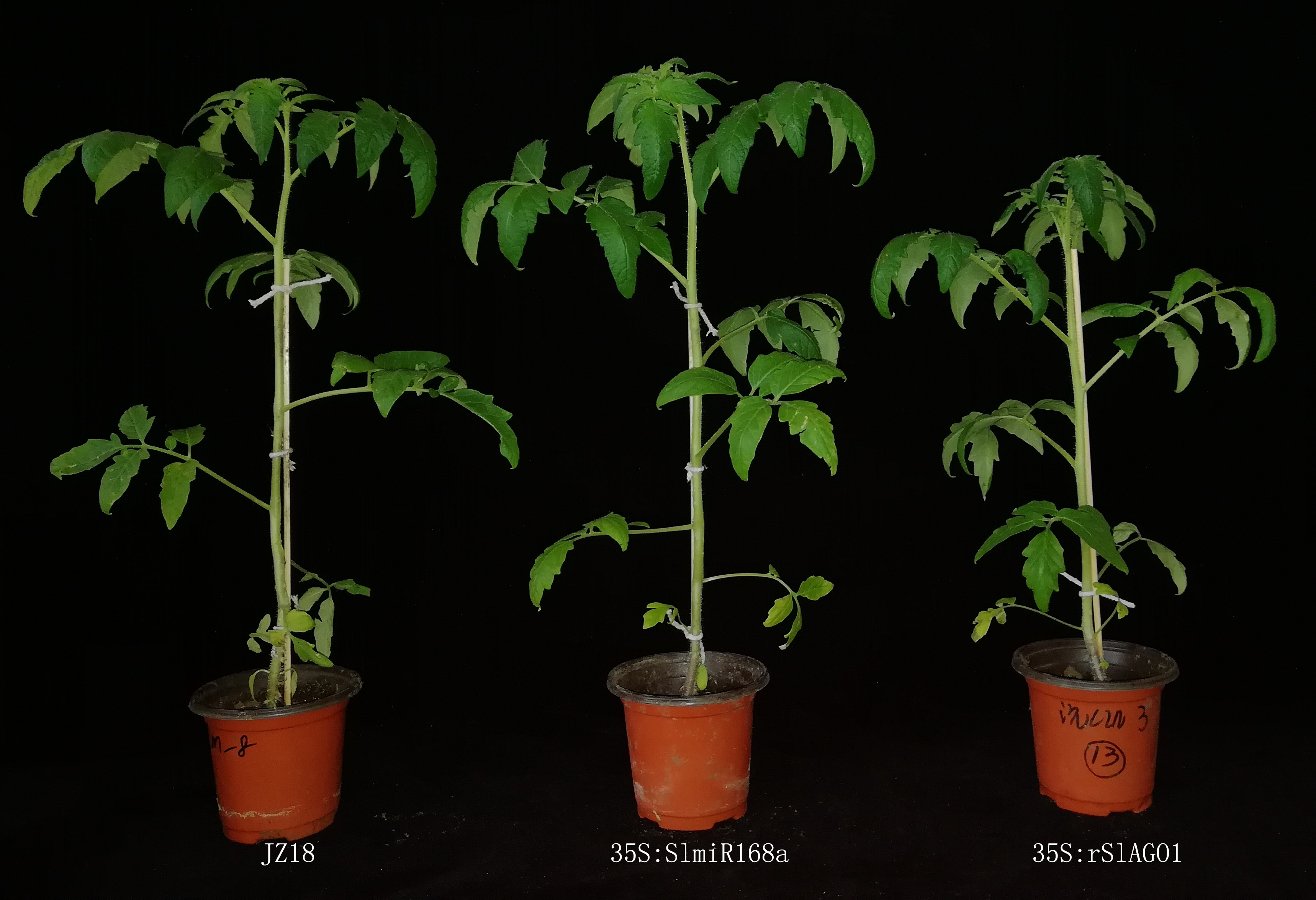

Supplement: Supplementary file 22 — Additional file 22: Figure S9. The phenotype of the whole plants of JZ18, 35S:SlmiR168a and 35S:rSlAGO1 under the normal condition. [file 12870_2020_2660_MOESM22_ESM.tif]
